# Copy number and sequence variation in rDNA of *Daphnia pulex* from natural populations: insights from whole-genome sequencing

**DOI:** 10.1093/g3journal/jkae105

**Published:** 2024-05-21

**Authors:** Abir Elguweidi, Teresa Crease

**Affiliations:** Department of Integrative Biology, University of Guelph, Guelph, ON, N1G2W1, Canada; Department of Integrative Biology, University of Guelph, Guelph, ON, N1G2W1, Canada

**Keywords:** *Daphnia pulex*, ribosomal RNA gene, copy number variation, intragenomic rDNA polymorphism, concerted evolution, polymorphic nucleotide sites

## Abstract

Ribosomal DNA (rDNA) has a vital role in ribosome biogenesis as it contains the genes that encode ribosomal RNA (rRNA) separated by intergenic spacers (IGSs). The rRNA genes occur in hundreds to tens of thousands of copies per haploid genome in eukaryotes and are generally highly conserved with low variation within species. Due to the repetitive nature and large size of rDNA arrays, detecting intraindividual variation can be difficult. In this study, we use whole-genome sequences of 169 *Daphnia pulex* individuals from 10 natural populations to measure the copy number and sequence variation in rDNA. This revealed that variation in rDNA copy number between individuals spans an order of magnitude. We further observed a substantial level of sequence variation within individual genomes. As expected, single-nucleotide polymorphisms occurred in regions of lower functional constraint such as the IGS and expansion segments of the rRNA genes. The presence of strong linkage disequilibrium among variants facilitated identification of haplotypes within each population. Although there was evidence of recombination among haplotypes from different populations, it is insufficient to eliminate linkage disequilibrium within populations. Estimating copy number and haplotype diversity within individuals revealed that the level of intraindividual sequence variation is not strongly correlated with copy number. The observed patterns of variation highlight a complex evolutionary history of rDNA in *D. pulex*. Future research should explore the functional implications of rDNA copy number and sequence variation on organismal phenotypes.

## Introduction

Ribosomal DNA (rDNA) encodes ribosomal RNA (rRNA) and is one of the best-characterized multigene families (Weider *et al*. 2005; Eickbush and Eickbush 2007). The importance of rDNA is not limited to its vital role in ribosome biogenesis as it has additional functions such as gene expression regulation and titrating chromatin (Paredes and Maggert 2009; Paredes *et al*. 2011; Larson *et al*. 2012; Gibbons *et al*. 2014). In eukaryotes, rDNA is typically organized in long tandem repeats (Venema and Tollervey 1999; Symonová 2019). Each repeated transcribed unit consists of the coding regions for18S, 5.8S, and 28S rRNA separated by two internal transcribed spacers (ITS1 and ITS2) and the external transcribed spacer. Moreover, each transcribed unit is separated by an intergenic spacer (IGS), which is downstream of the 28S rRNA gene (Venema and Tollervey 1999; Weider *et al*. 2005; Symonová 2019).

The rRNA coding sequences consist of core regions interspersed with expansion segments (Gerbi 1996). The core regions are highly conserved among eukaryotic organisms and generally homogeneous within species. However, the expansion segments and the noncoding spacer regions evolve more rapidly than the core regions and can vary substantially between even closely related species (Li *et al*. 2016; Guo *et al*. 2018). In addition, there is often intraspecific and intraindividual variation among repeat units (Ganley and Kobayashi 2007; Stage and Eickbush 2007; Huang *et al*. 2017).

The homogeneity of repeat units within species is a consequence of concerted evolution where the members of a gene family do not evolve independently (Nei and Rooney 2005; Weider *et al*. 2005; Eickbush and Eickbush 2007). The mechanism behind concerted evolution is thought to be recombination events that involve unequal crossovers and gene conversions, which can result in high sequence identity between units (Eickbush and Eickbush 2007; Ohta 2013). However, recombination events such as interchromosomal, intrachromosomal, and intrachromatid exchange as well as gene conversion between repeats along the rDNA array can also create variation in copy number among individuals within species (Eickbush and Eickbush 2007, Kobayashi 2011). For example, intraspecific variation in rDNA copy number has been documented in species such as *Daphnia* (Eagle and Crease 2012; LeRiche *et al*. 2014), mice (Gibbons *et al*. 2014), *Drosophila* (Paredes *et al*. 2011), fungi (Ganley and Kobayashi 2007; Simon and Weiß 2008), ciliates (Wang *et al*. 2017), and yeast (James *et al*. 2009).

Variation in rDNA copy number has been associated with phenotypic diversity, cancer development, and the aging process (Hall *et al*. 2022). In *Drosophila*, dramatically reduced copy number impacts protein synthesis (Xu *et al*. 2017; Sultanov and Hochwagen 2022). Morton *et al*. (2023) found that copy number below that typically found in wild-type strains of *Caenorhabditis elegans* affected development time and fertility. In addition, it is well established that rDNA copy number variation is affected by genetic and environmental factors (Nelson *et al*. 2019). For instance, Hosgood *et al*. (2019) demonstrated that copy number can be altered in the presence of bisphenol A in humans, whereas Ide *et al*. (2013) identified some genetic factors that affect copy number in budding yeast such as Rtt109, which plays a role in governing rDNA amplification. Despite this research, the relationship between rDNA copy number variation and phenotype is still poorly understood.

*Daphnia* is a small crustacean zooplankter in the order Cladocera (water fleas) inhabiting freshwater lakes and ponds on every continent except Antarctica. They are a keystone species in aquatic food webs (Miner *et al*. 2012) and known to be an important model organism in ecology and evolution studies (Stollewerk 2010). Most species reproduce by cyclical parthenogenesis in which direct-developing summer eggs are produced via apomixis during favorable environmental conditions. When conditions deteriorate (e.g. habitats dry up or freeze), diapausing eggs are produced via meiosis and require fertilization by sperm. In some species, meiosis has been lost and the diapausing eggs are also produced apomictically. Populations inhabiting temporary bodies of water must be re-established each year from diapausing eggs.

Studies of rDNA in *Daphnia* have provided some observations on the level of variation in copy number and sequence polymorphism across rDNA. For instance, studies of rDNA in individuals from several species in the *Daphnia pulex* species complex (Colbourne and Hebert 1996) based on restriction site analysis (Crease and Lynch 1991) and sequencing of cloned rDNA repeats (Crease 1995; McTaggart and Crease 2005; Ambrose and Crease 2011) found intraindividual sequence variation, while Eagle and Crease (2012, 2016) and LeRiche *et al*. (2014) reported variation in rDNA copy number in *D. pulex* and *Daphnia obtusa* using quantitative PCR (qPCR).

To our knowledge, the relationship between the level of intraspecific and intraindividual sequence polymorphism and copy number variation in eukaryotic rDNA has not been extensively studied. Therefore, we use whole-genome sequences of 169 *D. pulex* individuals from 10 natural populations in the Midwest United States and eastern Canada to determine levels of rDNA copy number and sequence variation to address the following questions: (1) is the level of intraindividual rDNA sequence variation correlated with rDNA copy number? (2) how is sequence variation distributed across the rDNA repeat? and (3) how much rDNA sequence variation is there within individuals and between individuals within and between populations?

## Materials and methods

### *D. pulex* genome data

Whole-genome sequences of 169 *D. pulex* individuals from 10 natural populations (Supplementary Table 1 in File S1) were obtained from the NCBI Sequence Read Archive [BioProjects PRJNA482684 (Maruki 2018) and PRJNA513203 (Ye 2019)]. These populations reproduce by cyclical parthenogenesis and are re-established from diapausing eggs each spring (Maruki *et al*. 2022). As *Daphnia* are very small animals, females sampled from nature were cultured in isolation and allowed to reproduce clonally for one to two generations. DNA for sequencing library preparation was extracted from a pool of each individual's daughters and granddaughters (Maruki *et al*. 2022).

We randomly chose up to 40 genomes that were at least 1.5 GB in size from each population. However, some had to be discarded as either one or both the variant and read depth analyses failed. This left from 14 to 24 individuals from each population. Each genome was stored in two FASTQ files containing the reverse and forward reads. Trimmomatic v 0.39 (Bolger *et al*. 2014) was used to remove adaptors. The trimmed genomes were aligned with the reference sequences of four rDNA regions and 16 exons from single-copy genes (SCG) using BWA mem v 0.7.17 (Li and Durbin 2009). Samtools v 1.17 (Danecek *et al*. 2021) was used to convert the output SAM files to sorted BAM files. Duplicate sequences were removed using Picard v 2.23.2 (Broad Institute, GitHub Repository, 2019) based on the default settings. Bedtools v 2.30.0 (Quinlan and Hall 2010) was used to calculate the per-base read depth of each reference sequence in the deduplicated BAM files. A summary of the read depth outputs (mean, mode, and median) was obtained using stats2.sh (McElroy *et al*. 2021).

### Reference sequence for rDNA regions

The rDNA repeat was separated into four regions: the two rRNA genes, 18S (2,293 bp) and 28S (4,376 bp), IGS unique region 1 (571 nt) upstream of the subrepeat region (Crease 1993), and IGS unique region 2 (3,123 nt) downstream of the subrepeat region. The IGS subrepeats were removed as they cannot be distinguished from one another using short-read sequence data. Sequences were obtained from GenBank and included the *D. pulex* 18S rRNA gene (accession no. AF014011; Colbourne *et al*. 1998), the *Daphnia pulicaria* 28S rRNA gene (accession no. AF346514; Omilian and Taylor 2001), and a *D. pulex* IGS sequence (accession no. L07948.1; Crease 1993). The rDNA reference sequences are available in File S2.

### Reference sequences for SCG exons

The Ensembl Metazoa 52 database and *D. pulex* genes (V1.0) dataset in Ensembl BioMart (https://metazoa.ensembl.org/Daphnia_pulex_gca021134715v1rs/Info/Index) were used to identify *D. pulex* protein-coding genes. We chose the following filters: homologs, *D. pulex* paralogs*, D. pulex* paralogs gene stable ID, and homology type as the criteria for searching. Using these criteria, we found 12,295 SCG. R (R Core Team 2021) was used to identify genes that occur in both the 12,295 SCG and the 716 single-copy orthologs conserved across eukaryotic genomes as described in Colbourne *et al*. (2011). This comparison reduced our list to 256 SCG.

A general transfer format file containing all information needed to extract the exons from the SCG was downloaded from the Ensembl Metazoa database, and Linux was used to create a list of exons in the SCG. A Python code was created to identify exons from 300 to 1500 bp, which resulted in 228 exons. We retained the longest exon from each gene, which reduced the number to 167 (Supplementary Table 1 in File S1). To generate a set of exons that consistently gave similar read depths within samples, we chose the genomes of 20 individuals from natural populations of *D. pulex* (Supplementary Table 1 in File S1) and 8 samples from *D. pulex* mutation accumulation lines (MAL; Harvey *et al*. 2020). These 8 MAL samples included 4 samples from a clonal hybrid lineage and 4 samples from a sexual lineage. The genome sequences of these 28 samples were mapped to the 167 exon sequences as indicated above. One individual from the natural populations was excluded from further analysis as the output file of exon read counts contained only zeros, resulting in a sample size of 27 genomes. We measured the interquartile range (IQR) of mean read depth for the 167 exons in each genome and then counted the number of genomes in which an exon fell within the IQR. This identified a set of 16 exons that fell within the IQR in at least 20 of the 27 genomes (74%). The difference between the mean read depth of these 16 exons and the mean read depth of all exons within the IQR of a genome was <2 reads in all cases. We also compared the read depth of the 16 exons in 50 genomes to the distribution of read depth in 160 of the 167 exons identified above. Read depth of seven exons was extremely high in some of these genomes, so these seven were excluded from this analysis (Supplementary Table 1 in File S1). The results are provided in File S3. Based on these analyses, we concluded that the 16 exons we chose provide an acceptable estimate of read depth of SCGs and we used them to estimate haploid copy number of the four rDNA regions. The exon reference sequences and ID numbers are available in File S2.

### Estimating copy number of rDNA

Genome sequences of the 169 individuals were mapped to the four rDNA and 16 exon reference sequences, and the per-base and mean read depth of each reference sequence was estimated as indicated above. The copy number of each rDNA region was estimated by dividing the read depth of the rDNA region by the mean read depth of the 16 exons. Because this ratio gives the number of rDNA reads per SCG read, it provides an estimate of haploid rDNA copy number. To obtain the diploid rDNA copy number for the heterozygosity analysis and analysis of molecular variance (AMOVA) (see below), we multiplied our estimates by 2.

To determine the correlation of copy number between rDNA regions and to determine the slope of the line of best fit, we performed a regression analysis in R based on our estimates of haploid copy number. Although no rDNA region is a dependent variable relative to any other region, if there is a significant correlation, we should be able to predict the copy number of one rDNA region based on the copy number of another region. In the case of rDNA, we expect the slope of a regression line between any two regions to be 1 if each rDNA repeat unit contains only one copy of each rDNA region, and these sequences do not occur outside the rDNA array.

### Variant calling

Bcftools v 1.11 was used to identify single-nucleotide polymorphisms (SNPs) in the rDNA regions by creating an mpileup file for each genome. The mpileup files were manipulated using R (R Core Team 2021) to generate summaries of the read depth of the mapped reads for each of the four nt. Allele 1 was always the nt in the reference sequence. Allele counts lower than 5 at a SNP in a sample were set to 0, and a new total read depth for that site was calculated. SNPs with a total read depth <99 in a genome were discarded. Allele frequency at each SNP in each sample was obtained by dividing the read count for each nt by the total read depth of all 4 nucleotides. A SNP was excluded from further analysis if the mean frequency of allele 1 (the reference allele) was more than 0.990 across all genomes within a population. We calculated the number of SNPs and their distribution across the dataset. Additionally, we determined the frequency of each SNP. The localization of SNPs in the 28S gene was achieved by aligning the *D. pulex* sequence with the *Drosophila melanogaster* sequence (accession no. M21017.1). The location of helices in the *D. melanogaster* sequence, as identified by Van de Peer *et al*. (2000), was used for this alignment.

### Identifying rDNA haplotypes

Many of the SNPs were close to one another, and initial inspection of the data indicated that there was substantial linkage disequilibrium between sites. In addition, the list of SNPs varied among populations. Thus, we analyzed SNP allele frequencies in each population separately using the R package, haploSep (Pelizzola *et al*. 2021), to identify haplotypes and their frequencies in each individual. Before running haploSep, we used haploSelect to determine the number of haplotypes to expect in each population. However, if this number exceeded the log2 of the number of SNPs, we used the smaller number in haploSep as suggested by Pelizzola *et al*. (2021). In addition, the “bias’ parameter was set to FALSE so that haplotype frequencies within individuals would sum to 1, which means that minor haplotypes including low-frequency variants may not have been identified. However, using these two settings gave identical results in multiple runs of the program.

To evaluate the relationship among haplotypes based on the 222 SNPs identified across all populations, a network based on the number of differences between haplotypes was generated using the Templeton, Crandall, and Sing (TCS) method (Templeton *et al*. 1992) and the program, PopArt (https://popart.maths.otago.ac.nz/).

To explore the relationship between rDNA copy number and haplotype variation, the expected heterozygosity (H_e_) for each individual was calculated as 1—(∑*p_i_*^2^) where *p_i_* is the frequency of *i*th haplotype in an individual. A linear regression between H_e_ and diploid 28S copy number, obtained by multiplying the haploid estimate by 2 was then performed in R.

### Analysis of molecular variance

A hierarchical AMOVA based on the genetic distance between haplotypes and their abundance in each of the 169 individuals was performed using Arlequin v 3.5.2.2 (Excoffier and Lischer 2010). Genetic distance between haplotypes was calculated as the pairwise proportion of differences at the 222 SNPs. The sample size of rDNA repeats within individuals was based on the diploid number of 28S genes. The three hierarchical levels were rDNA variation within individuals, variation between individuals within populations, and variation between populations. These values were used to estimate the fixation indices *F*_SC_ (genetic differentiation among individuals within a population) and *F*_CT_ (genetic differentiation among populations). We also estimated the proportion of variation within and among individuals for each population separately. Significance was tested by generating 1,000 random permuted samples.

Arlequin was also used to calculate the pairwise genetic distance between all individuals based on the number of differences between haplotypes and their relative abundance. The R package, ape (https://github.com/emmanuelparadis/ape) was used to generate a neighbor-joining (NJ) tree (Saitou and Nei 1987) from the genetic distance matrix. The distance values were very skewed, so the square root of each value, which compresses high values and spreads out the low values, was used to generate the NJ tree.

### Mantel test

A Mantel test was performed in Arlequin to evaluate the relationship between genetic divergence and geographic distance among the 10 populations. All individuals within a population were combined for this analysis. Pairwise *F*_CT_ values were calculated from the proportion of nucleotide differences between haplotypes and their abundance within populations calculated from the diploid number of 28S genes. The Mantel test was based on the correlation between the matrix of linearized *F*_CT_ values [*F*_CT_/(1−*F*_CT_)] and geographic distance between populations. Significance was tested by generating 1,000 random permuted samples. A NJ tree of the pairwise *F*_CT_ values was generated using MEGA11 (https://www.megasoftware.net/; Tamura *et al*. 2021).

## Results

### Haploid copy number of rDNA

**Fig. 1.**
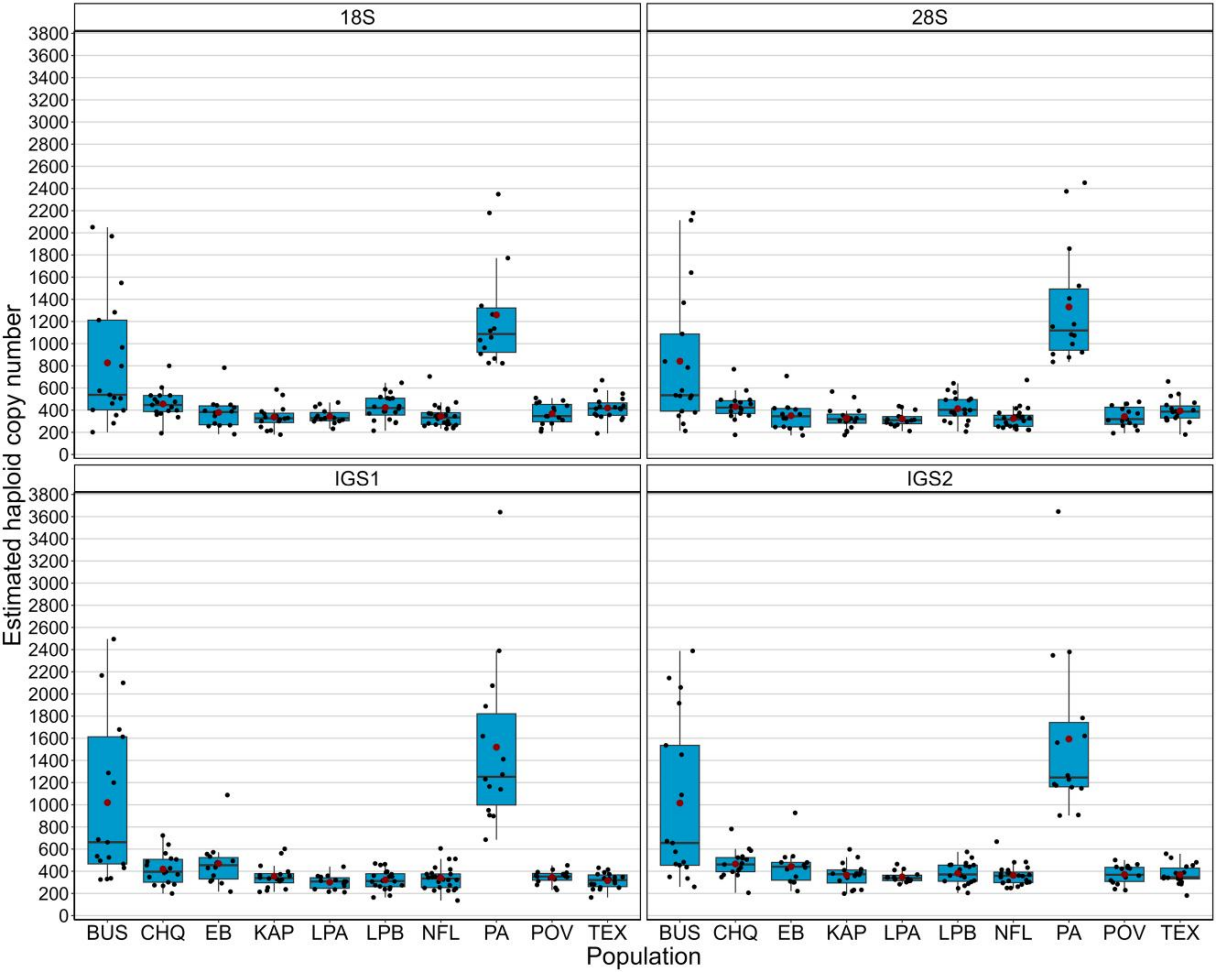
Haploid copy number of four rDNA regions (18S, 28S, IGS1, and IGS2) in 169 *Daphnia pulex* individuals from 10 populations in eastern United States and Canada. The box indicates the IQR, the whiskers show the 1.5*IQR values, the horizontal line indicates the median, and the red dot indicates the mean.

**Fig. 2.**
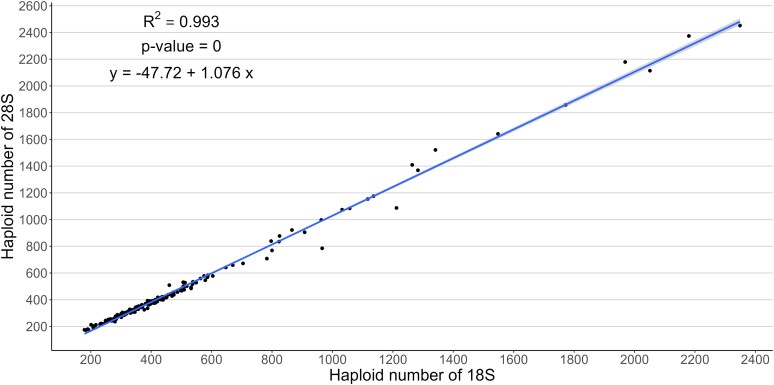
Linear regression of haploid copy number of 28S and 18S rRNA genes in 169 *D. pulex* individuals from 10 populations. The shading on either side of the line indicates the 95% confidence interval.

The haploid copy number of four rDNA regions in 169 individuals from 10 natural populations of *D. pulex* varied from 179 to 2,349 for the 18S gene, 173 to 2,452 for the 28S gene, 164 to 3,640 copies for IGS1, and 903 to 3,645 copies for IGS2 (Fig. 1, Supplementary Table 2 in File S1). Individuals from PA and BUS exhibited higher rDNA copy number, on average compared to other populations (Fig. 1, Supplementary Table 3 in File S1). Further, the number of 18S and 28S genes was strongly correlated (*P*-value < 0.001) with an *R*^2^ = 0.993 (Fig. 2) and a regression coefficient slightly higher than 1 (1.08). The regression analysis of haploid copy number of IGS2 on IGS1 showed that they are also highly correlated (*R*^2^ = 0.981, *P*-value < 0.001; Supplementary Fig. 1 in File 4) but not as strongly as the two rRNA genes. When the spacers were regressed on the two genes: IGS1 with 28S (*R*^2^ = 0.891) and IGS2 with 18S (*R*^2^ = 0.936), the correlation was significant but again, not as strong as the correlation between the rRNA genes (Supplementary Figs. 2 and 3 in File S4).

Most haploid 28S gene copy numbers (73%) fell between 200 and 500 with only 15% greater than 600 (Supplementary Fig. 4 in File S4). Most of the individuals with high copy number came from BUS (7 of 17) and PA (all 14), with one each from CHQ, EB, LPB, NFL, and TEX.

### rDNA variation

**Fig. 3.**
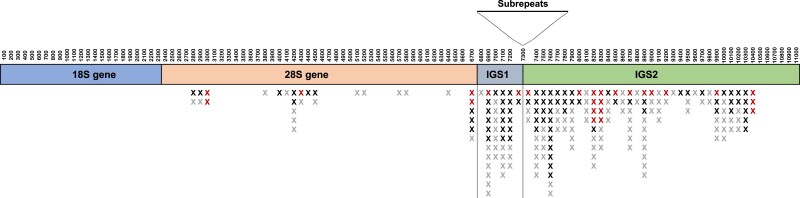
Distribution of 222 SNPs in the rDNA repeat of 169 *D. pulex* genomes from 10 populations. The rDNA repeat unit is divided into 100 nt windows. The subrepeat region between the intergenic spacers (IGS1 and IGS2) was omitted from analysis. This region is composed of a variable number of three subrepeat types: subrepeat a (184–222 nt), subrepeat b (96–100 nt), and subrepeat c (184–194 nt) (Ambrose and Crease 2011). Red Xs (26) indicate sites that were polymorphic in 8, 9, or 10 populations. Gray Xs (117) indicate sites that were polymorphic in only one population.

There were 222 SNPs that met our criteria for retention in the 169 *D. pulex* individuals (Table 1). The reference allele was the most common allele at all SNPs. There were only two alleles at most sites, but third and fourth alleles were also observed in some individuals (Supplementary Table 2 in File 1). As expected, most SNPs were in the spacers with 150 in IGS2, 42 in IGS1, and 30 in the 28S gene (Fig. 3). Most SNPs in the 28S gene were in variable regions as defined by Van de Peer *et al.* (2000) with 23 in V2, V6, V8, and V12. Seven SNPs were outside variable regions: one occurred in helix D17, two in helix D22, one in helix G5, one between helices G5-1 and G5-2, one between helices H4 and H1, and one downstream of helix E9.

**Table 1.** Distribution of rDNA sequence variation in 169 *D. pulex* individuals from 10 natural populations.

Population	Number of SNPs	Number of haplotypes	Mean difference between haplotypes (nt)	Lowest number of differences between haplotypes (nt)	Highest number of differences between haplotypes (nt)
BUS	29	4	13.7	4	24
CHQ	48	5	22.0	7	35
EB	71	6	24.6	12	36
KAP	67	6	27.0	6	43
LPA	86	6	33.9	5	54
LPB	85	6	34.0	7	65
NFL	79	6	34.7	7	56
PA	52	5	22.6	6	38
POV	60	5	26.6	8	44
TEX	70	6	22.2	4	43
Overall	222	55*	29.1	0	81

^*^There are 54 unique haplotypes. Haplotypes LPA2 and POV1 are identical.

**Fig. 4.**
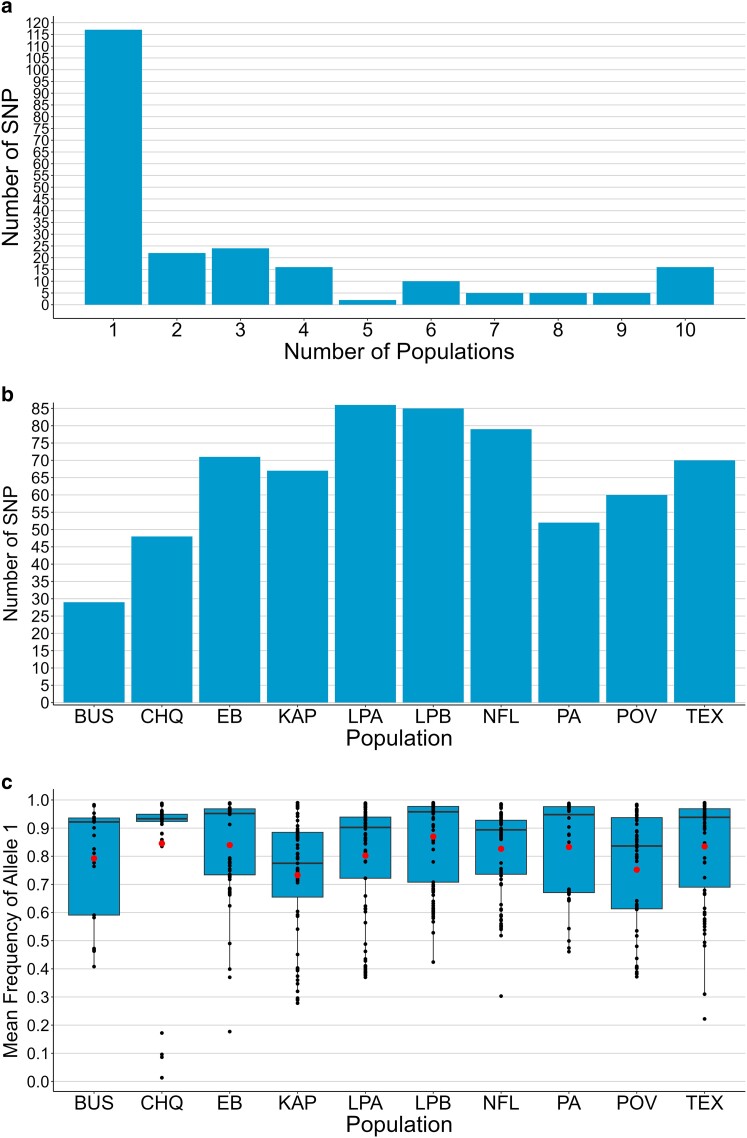
Distribution and mean frequency of 222 SNPs in the rDNA of 169 *D. pulex* individuals from 10 populations. a) Distribution of SNPs across populations. For example, 117 SNPs occur in only one population. b) Number of SNPs in each population. c) Mean frequency of allele 1 across all SNPs within an individual and across all individuals within a population.

We calculated the distribution and number of SNPs in each population and found that 26 (12%) of the 222 SNPs were present in 8, 9, or 10 populations (Fig. 4a, Supplementary Table 4 in File S1), with 2 in IGS1, 5 in the 28S gene, and 19 in IGS2 (Fig. 3). Meanwhile, 117 (53%) of the 222 SNPs were unique to a single population (Fig. 4a), with 21 in IGS1, 15 in the 28S gene, and 81 in IGS2.

SNPs found in only two or three populations were not always found in the populations closest to one another (e.g. IGS2_0016 in EB and PA, IGS2_1126 and IGS2_2732 in EB and TEX, r28S_0473 in EB and NFL, and IGS2_3024 in EB, LPA, and TEX, Supplementary Table 4 in File 1). Additionally, the number of SNPs in each population varied from 29 in BUS to 86 in LPA (Table 1, Fig. 4b). The mean frequency of allele 1 (the reference allele) across SNPs varied substantially among individuals within a population and among populations (Fig. 4c). In some individuals, the value was below 50% (Supplementary Table 4 in File S1), indicating that variant alleles predominated at some SNPs.

**Fig. 5.**
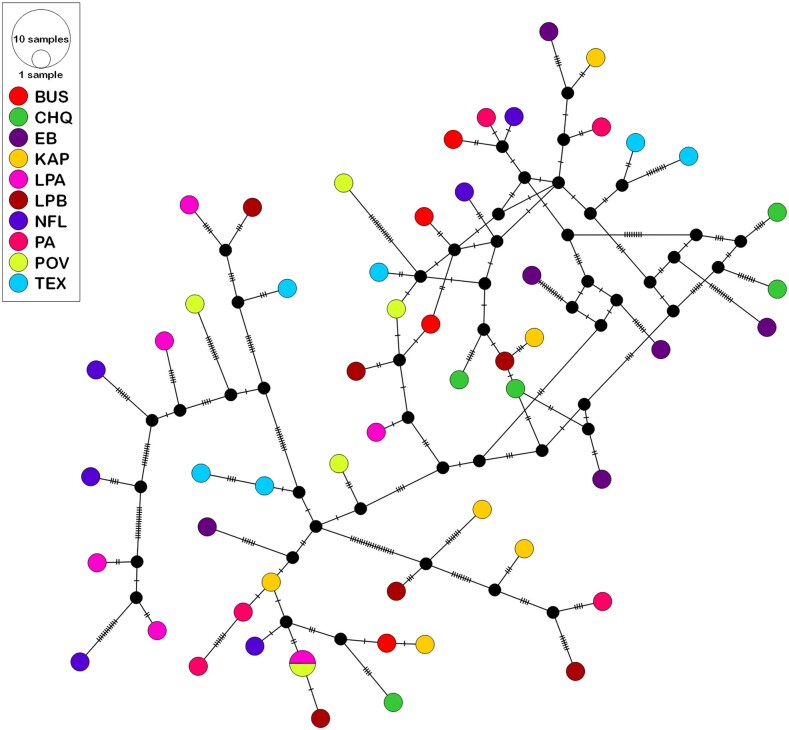
TCS haplotype network of the relationship among the 55 rDNA haplotypes identified in 169 *D. pulex* individuals from 10 populations. Hatch marks along the branches indicate the number of nucleotide differences between nodes.

**Fig. 6.**
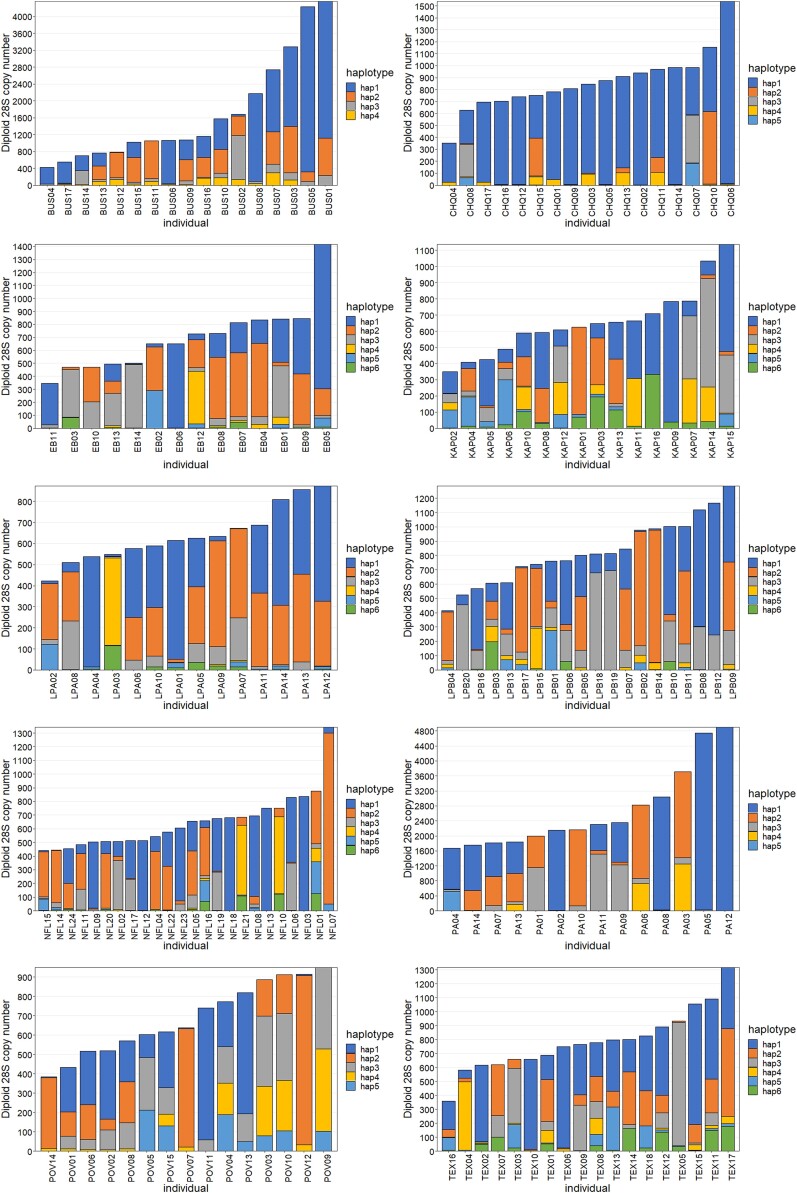
Counts of the 55 rDNA haplotypes in 169 *D. pulex* individuals from 10 populations. Individuals in each population are ordered according to the diploid copy number of 28S genes. Although the same colors are used in each plot, each population has different haplotypes. The only haplotype shared between populations is LPA2, which is the same as POV1.

HaploSep detected 55 haplotypes in the 169 individuals (Fig. 5). Their genotype at all 222 polymorphic sites is provided in File S5. Only one haplotype was found in more than one population: haplotype 2 in LPA and haplotype 1 in POV (Fig. 5). Pairwise differences between all other haplotypes ranged from 1 nt to 81 nt. A TCS network of the 55 haplotypes (Fig. 5) shows that the haplotypes do not cluster by population and there has been substantial recombination between them. Each population had a minimum of four and a maximum of six haplotypes (Table 1). The maximum number of differences between two haplotypes in the same population was 65 (LPB). The average number of differences between haplotypes within a population ranged from 13.7 (BUS) to 34.7 (NFL). Individuals often did not contain all the haplotypes found in their population (Fig. 6, Supplementary Table 2 in File 1). In addition, the dominant haplotype differed among individuals as did the relative frequency of haplotypes (Fig. 6).

**Fig. 7.**
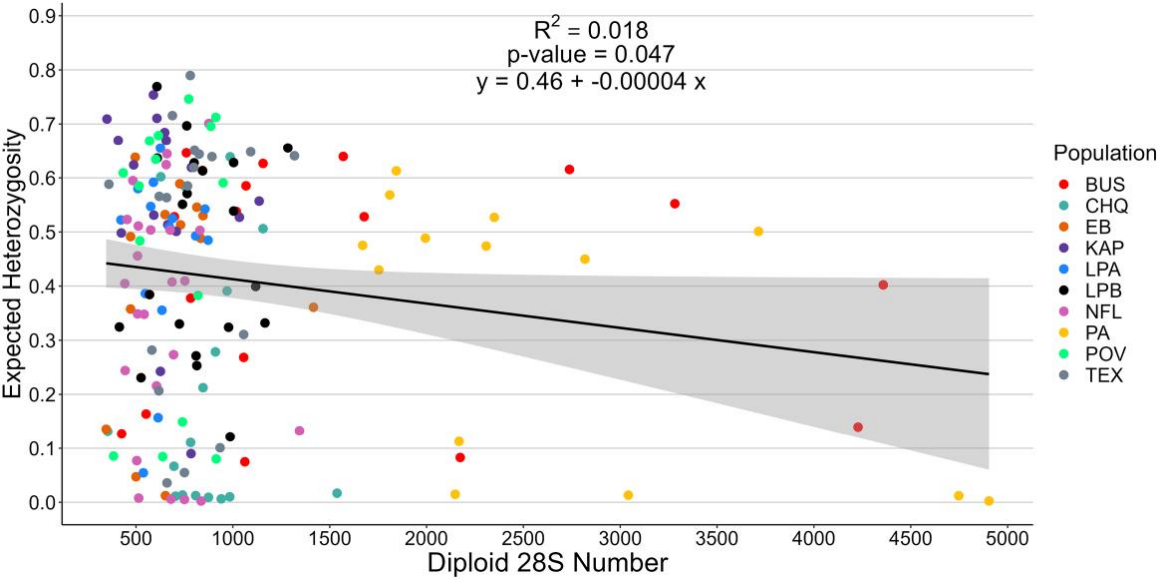
Regression of expected haplotype heterozygosity on the diploid copy number of 28S genes in 169 *D. pulex* individuals from 10 populations. The shading on either side of the line indicates the 95% confidence interval.

A regression of the H_e_ within individuals and their diploid number of 28S genes was significant (*R*^2^ = 0.018, *P* = 0.047) with a negative slope of −0.00004, suggesting that large rDNA arrays tend to have somewhat less variation than smaller ones (Fig. 7). Indeed, expansion of diploid copy number within individuals tended to be associated with an expansion of one haplotype, for example, haplotype 1 in BUS01 and BUS05, and haplotype 2 in EB05 (Fig. 6). On the other hand, when individuals from BUS and PA, many of which had unusually high copy number, were omitted from the analysis, there was no correlation (*R*^2^ = 0.002, *P* = 0.602), so overall, the level of haplotype variation within an rDNA array was not strongly influenced by its size unless copy number was unusually large.

**Fig. 8.**
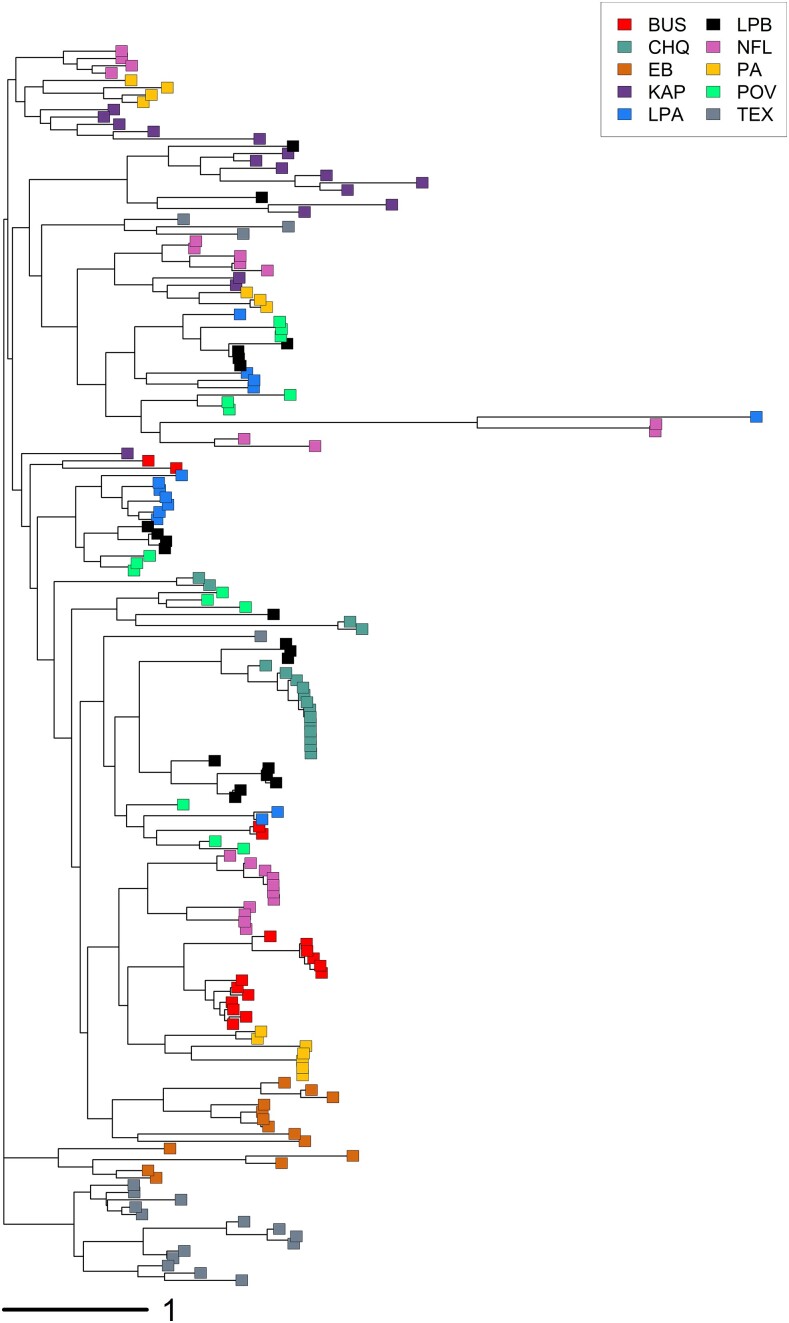
A NJ tree generated from square root–transformed pairwise genetic distances between 169 *D. pulex* individuals from 10 populations. Distances are based on the number of nucleotide differences between haplotypes and their abundance within individuals based on diploid 28S copy number. Only the 222 SNPs were included in this analysis.

Because the distribution of genetic distance values based on the number of differences between haplotypes and their frequency between all pairs of individuals was highly skewed to the left, we square root–transformed the values. The NJ tree generated from the transformed matrix of pairwise genetic distance values (Fig. 8) shows that individuals within populations tend to cluster together, although this is not always the case.

### Analysis of molecular variance

The AMOVA (Table 2) indicated that the percentage of variation within individuals was higher than the variation among individuals in all populations except NFL and PA. Moreover, *F*_SC_ values of differentiation among individuals within populations varied from 0.305 (TEX) to 0.623 (NFL). Overall, intraindividual variation among individuals within populations was higher (*F*_SC_ = 0.459) than the level of variation between populations (*F*_CT_ = 0.253).

**Table 2.** Results of AMOVA of rDNA variation in 10 natural populations of *D. pulex*.

Population	Number of individuals	Percent variation within individuals	Percent variation among individuals within populations	Percent variation among populations	*F*_SC_
BUS	17	68.05	31.95		0.320
CHQ	17	59.29	40.71		0.407
EB	14	56.83	43.17		0.432
KAP	16	64.40	35.60		0.356
LPA	14	58.24	47.76		0.418
LPB	20	57.86	42.14		0.421
NFL	24	37.72	62.28		0.623
PA	14	36.25	63.75		0.638
POV	15	65.09	34.91		0.349
TEX	18	69.47	30.53		0.305
All populations	169	40.44	34.28	25.28	*F*_SC_ = 0.459 *F*_CT_ = 0.253

The *P*-value of all fixation indices is 0.00000.

**Fig. 9.**
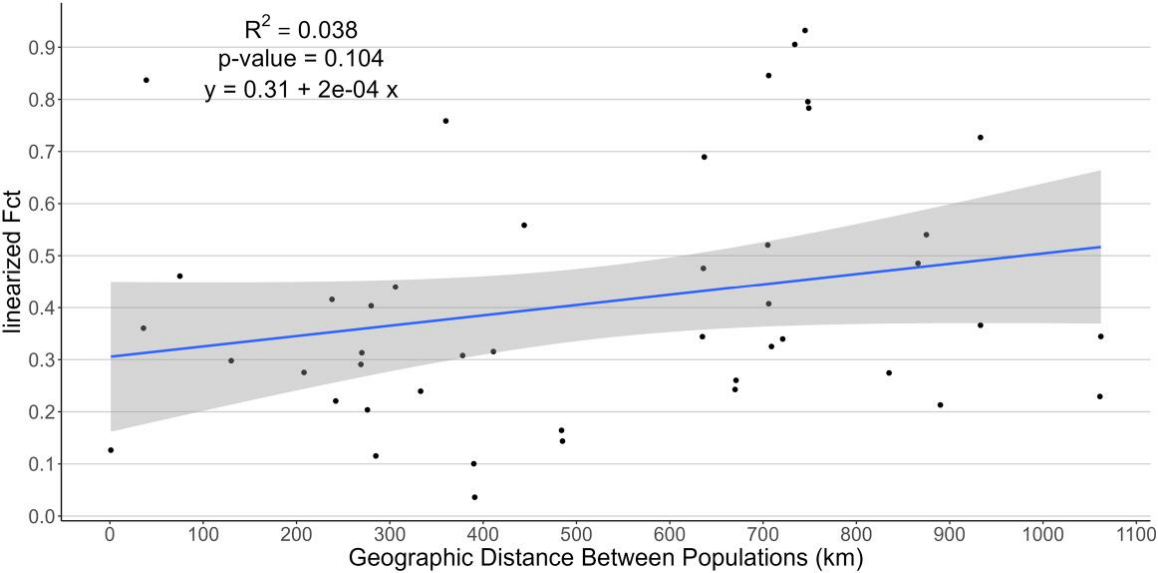
Regression of linearized pairwise *F*_CT_ values based on rDNA variation and geographic distance between 10 populations of *D. pulex* in eastern United States and Canada. The shading on either side of the line indicates the 95% confidence interval.

**Fig. 10.**
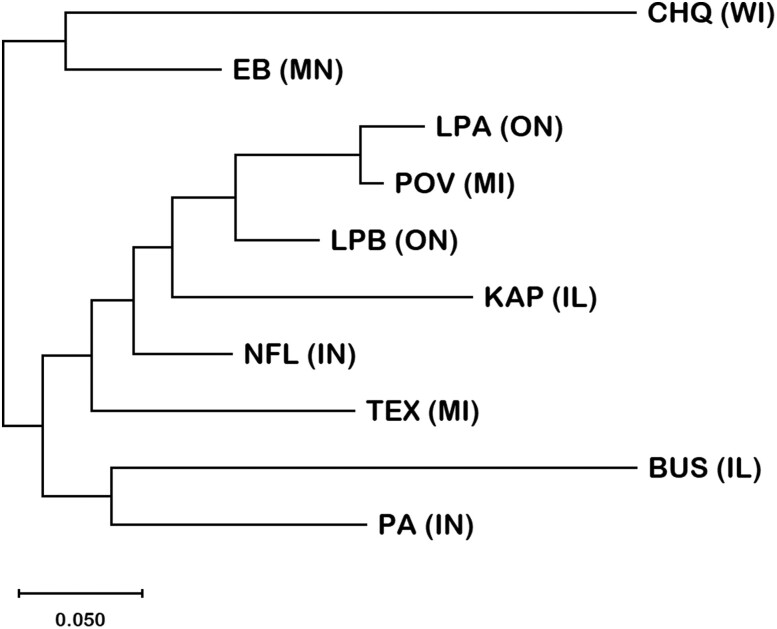
A NJ tree generated from pairwise *F*_CT_ values between the 10 populations of *D. pulex*. *F*_CT_ estimates are based on the number of nucleotide differences between haplotypes and their abundance within populations, which is based on total diploid 28S copy number of all individuals in a population. Only the 222 SNPs were included in this analysis.

The Mantel test based on pairwise linearized *F*_CT_ and geographic distance among the ten populations was positive but not significant (*R*^2^ = 0.038, *P*-value = 0.104), indicating that there was no significant isolation by distance between populations (Fig. 9). This is concordant with the NJ tree generated from the pairwise *F*_CT_ estimates (Fig. 10) in which populations closest to one another geographically do not always cluster with one another. For example, PA from Indiana and BUS from Illinois cluster together, but the other populations from these two states (KAP, NFL) cluster with the populations from Michigan (POV, TEX) and Ontario (LPA, LPB).

## Discussion

In this study, we investigated copy number and sequence variation in the rDNA of 169 individuals of *D. pulex* from 10 natural populations and observed substantial variation in both components. Research on rDNA sequence variation has focused on concerted evolution, which is the process that maintains high sequence identity among rDNA repeats within a genome and across arrays in a species. It is important to note that concerted evolution is not an absolute process; a certain level of heterogeneity is expected between repeat units because it may take a long time for a new variant to become fixed in a population and then the entire species. The rate of fixation of variants is associated with the rate of recombination, the size of the gene family, and population-level processes such as genetic drift, gene flow, and natural selection (Elder and Turner 1995; Liao 1999; Eickbush and Eickbush 2007). Transposable element insertions in rDNA arrays, which are known to occur in *D. pulex* (Glass *et al*. 2008), the presence of multiple chromosomal locations for rRNA gene arrays, and epigenetic effects such as methylation and gene silencing are other mechanisms believed to restrict the process of concerted rDNA evolution within a species (Bik *et al*. 2013), including *D. pulex* (Glass *et al*. 2008). Transposable elements might also be responsible for variation in the arrangement and quantity of rRNA genes within a species (Veltsos *et al*. 2009). For example, Lim *et al*. (2000) showed that the act of silencing rRNA genes through methylation or heterochromatization could markedly inhibit the process of concerted evolution.

### rDNA copy number variation

The substantial variation we observed in rDNA copy numbers among individuals both within and among populations highlights the dynamic nature of rDNA copy number within a species. We observed higher haploid copy numbers in BUS (the 18S mean copy number is 827 and ranges from 201 to 2051) and PA (the 18S mean is 1,260 and ranges from 823 to 2349) compared to the other populations in this study, indicating that the number of rRNA genes can differ as much as tenfold among individuals of the same species. Indeed, the lowest haploid 18S copy number in PA was 835, which exceeds the maximum estimate in all other populations except BUS. We checked the genome information provided in the Sequence Read Archive and could find nothing unusual about the 14 PA and 17 BUS genomes we chose to analyze. The libraries from BUS and PA were run on a HiSeq2500 on the same day. The libraries from EB were also run on a HiSeq2500 two days earlier. All other libraries were run on a NextSeq500. The sizes of genome sequences of the 14 PA and 17 BUS genomes were not unusually large or small compared to the other genomes from these populations. Moreover, nothing about patterns of genetic diversity at the genome level suggests that either BUS or PA is unusual (Maruki *et al*. 2022).

To determine whether the estimates in PA could be an experimental artifact, we downloaded and analyzed 15 PA genomes from another study (Ye *et al*. 2023, PRJNA684968). These libraries were all run on a HiSeq2500 over five days. Haploid 18S number varied from 221 to 1,696 (3 estimates were > 1000), which is closer to the values we obtained for the other populations, including BUS. Thus, it is not clear whether the estimates included in this study represent experimental artifacts or the actual rDNA copy number. The repeatability of the high estimates could be determined by making new libraries from the previously analyzed DNA samples and sequencing them to see whether the same results are obtained. If these estimates are valid, additional research will be required to determine the basis for the unusually high rDNA copy number in these two populations, and if it has phenotypic consequences.

The strength of the correlation between the two rRNA genes and a regression coefficient very close to 1 is expected if every rDNA repeat contains one copy of each gene and there are no copies outside the rDNA array. This correlation can serve as an indicator of the level of accuracy of the method that was used to estimate rDNA copy numbers. The slight deviation from a 1:1 ratio, with a regression coefficient slightly higher than 1, could be a result of experimental variation in read depth across all nucleotides in each gene.

The correlation between the IGS1 and IGS2 spacer regions was significant but less pronounced than that between the rRNA genes. Furthermore, the correlation between the genes and the spacers was not as strong as that between the genes or the spacers and the regression coefficients were greater than 1. The variation in correlation may arise from portions of the IGS being clustered at chromosomal locations outside the rDNA, deviating from their typical association with the genes (De Lucchini *et al*. 1988). However, this phenomenon has not been documented in *D. pulex*. Additionally, it is possible that the reduced correlation between rDNA regions could be an experimental artifact.

The range of our rDNA copy number estimates is higher than the estimates obtained in previous research using qPCR. For instance, Eagle and Crease (2012) estimated the copy number of rRNA genes in 43 individuals from 16 ponds of *D. pulex* and 26 individuals from 6 lakes of *D. pulicaria* including one individual from PA (haploid 18S = 180) and LPA (haploid 18S = 166), and two from LPB (haploid 18S = 124, 207). They found substantial differences in gene copy numbers between some individuals, with haploid 18S copy numbers ranging from 94 to 490 and similar values for the 28S gene. In a separate study, Eagle and Crease (2016) investigated rRNA gene copy number variation in 26 *D. pulex* isolates, including three from PA (haploid 18S = 133, 166, 173), and three from LPB (haploid 18S = 348, 397, 441); 5 *D. pulicaria* isolates, 4 *D. arenata* isolates, 8 European *D. pulex* isolates, and 2 European *D. pulicaria* isolates. The haploid 18S copy number ranged from 98 to 460 across all isolates, while it ranged from 90 to 577 for 28S. Although their estimates for PA are much lower than the ones from the 14 samples in our study, they are similar to those from the additional 15 individuals we analyzed. Moreover, the estimates for LPA and LPB are well within the range we observed (232 to 469 and 214 to 647, respectively). Moreover, the correlation between copy numbers of the rRNA genes based on qPCR was lower at 94%.

LeRiche *et al*. (2014) also observed substantial variability in the copy number of rRNA genes among 21 *D. obtusa* isolates sampled from 15 natural populations. Using qPCR, they estimated haploid 18S copy numbers from 190 to 871, with an average of 423, while for 28S, values ranged from 180 to 959, with an average of 405. Overall, the range of haploid rDNA copy number estimates in *Daphnia* obtained using qPCR and genome sequencing is similar, although to the best of our knowledge, estimates larger than 1,000 have not been obtained in *Daphnia* using qPCR, nor has anyone used both methods to analyze the same DNA samples from extremely high copy–number individuals.

Estimating rDNA copy number is a challenging task, and various methods have been employed for this purpose, each with its own set of complications. For instance, pulsed-field gel electrophoresis with contour-clamped homogeneous electric field (CHEF), followed by rDNA-specific Southern blotting and hybridization, is considered the optimal method for estimating rDNA copy number (Morton *et al*. 2020). However, this method is time-consuming and technically challenging, and the large size and complex structure of rDNA in organisms like humans make this technique less useful (Morton *et al*. 2020).

The use of qPCR is widespread, but it comes with some limitations that can make it difficult to obtain accurate copy number estimates. For example, primer biases can lead to either overestimating or underestimating the copy number of the target gene. Further, qPCR may reach a point of saturation beyond which copy number cannot be accurately measured. Therefore, it becomes less reliable for quantifying very large copy numbers. A related method for estimating rDNA copy number is digital droplet (dd)PCR. It offers improved resolution and replicability compared to traditional qPCR and has provided more accurate copy number estimates in yeast and human studies (Hall *et al*. 2022).

Short-read sequencing, which was used in the present study, is also commonly used to estimate rDNA copy number. However, differences in library preparation methods or conditions under which the sequencing data were generated may affect the results such that they do not reflect true biological differences (Hall *et al*. 2022). Morton *et al*. (2020) outlined several best practices to ensure the highest accuracy in estimating rDNA copy numbers such as using CHEF, when feasible, to validate copy numbers obtained from whole-genome sequencing. In addition, when using whole-genome sequencing or ddPCR, only samples that have been prepared and run together should be compared. However, this may not be feasible for large numbers of samples, in which case repeatability of estimates could be determined by making replicate sequencing libraries from some individuals on different days and sequencing them in different runs and/or on different instruments.

Intraspecific variability in rDNA copy numbers is not unique to *Daphnia* as such variation has been reported in numerous eukaryotic genomes including humans (Gibbons *et al*. 2014, 2015; Warmerdam *et al*. 2016), mice (Gibbons *et al*. 2015), nematodes (Bik *et al*. 2013; Thompson *et al*. 2013), freshwater snails (McElroy *et al*. 2021), and plants including barley (Zhang *et al*. 1990), bell bean (Rogers and Bendich 1987), and *Arabidopsis* (Zoschke *et al*. 2007). Furthermore, the range of rDNA copy number in some studies was as high or higher than the range we observed in the present study. For example, *D. melanogaster* exhibited a range of variation from 80 to 600 haploid copies (Mohan and Ritossa 1970) and 10-fold variation has been reported for both humans and mice (Gibbons *et al*. 2015).

Through computer simulations, Zhang *et al*. (2008) demonstrated that the size of the rDNA locus is primarily determined by the frequency of sister chromatid exchange, resulting in extensive variability among individuals and large rDNA loci. Conversely, elevated rates of interchromosomal exchange tend to decrease interindividual variation and reduce the overall size of the rDNA locus. This model suggests that the substantial interindividual variation in the number of rRNA genes seen in *D. pulex and D. pulicaria* arises from higher intrachromosomal exchange rates compared to interchromosomal exchange rates, consistent with earlier studies on rDNA variation in *Daphnia* (Crease 1995; McTaggart *et al*. 2007).

Recent studies have emphasized the impact of reduced rDNA copy numbers on various phenotypes. These reductions have been linked to global transcriptional changes, cancer, and aging (Paredes and Maggert 2009; Xu *et al*. 2017; Nelson *et al*. 2019). For example, significant reductions in rDNA copy number can lead to developmental issues in model organisms. In *C. elegans*, the complete removal of 45S rDNA results in a peculiar situation where worms complete embryonic development with maternal ribosomes but remain stuck in the initial larval stage (Cenik *et al*. 2019). Worms with substantial rDNA reductions compared to wild type were able to develop to adulthood, but both development time and fertility were negatively affected (Morton *et al*. 2023). In *D. melanogaster* (Nelson *et al*. 2019), having an insufficient number of functional rDNA copies can manifest as the classic “bobbed” phenotype, which results in tissue-specific defects. More extensive reductions in rDNA copy numbers can even be fatal. Likewise, in yeast, low rDNA copy number makes DNA more susceptible to mutations and can cause issues with DNA replication and cause loss of rDNA copies (Hall *et al*. 2022).

When rDNA copy number exceeds a certain threshold, the differences no longer significantly correlate with rRNA levels. This is because only 50% or even fewer rRNA genes are actively transcribed, as observed in humans (Gagnon-Kugler *et al*. 2009). In specific strains of *D. melanogaster*, as few as 10% of copies are transcriptionally active (Ye and Eickbush 2006). Moreover, Mohan and Ritossa (1970), who determined that rDNA copy number in *Drosophila* ranged from 80 to 600 copies, found that individuals with more than 130 copies did not exhibit any observable consequences related to the increased copy number. Similarly, Lopez *et al*. (2021) found no decrease in rRNA transcription rate in *Arabidopsis thaliana* lines whose rDNA copy number was reduced by 90% compared to wild-type individuals, and Li *et al*. (2018) found no correlation between rDNA copy number and levels of rRNA transcription in inbred lines of maize.

In contrast to the lack of association between rDNA copy number and levels of rRNA transcription, other researchers have found that rDNA copy number variation does affect levels of transcription of other genes in humans (Gibbons *et al*. 2014) and *Drosophila* (Paredes and Maggert 2009). In addition, the existence of extra, nontranscribed rRNA genes and the spacers that separate them has been shown to contribute to genetic stability by acting as anchor points for repair enzymes and facilitating connections with other parts of the genome (O'Sullivan *et al*. 2009; Ide *et al*. 2010; Cahyani *et al*. 2014). A study of rDNA on the *Drosophila* Y chromosome (Paredes and Maggert 2009) revealed that a reduction in rDNA decreases gene silencing in other genomic regions, and the more extensive the deletion of rDNA, the greater the reduction in gene silencing. Thus, changes in rDNA can influence gene expression at other loci by altering the balance between heterochromatin and euchromatin in the nucleus during development (Paredes and Maggert 2009). Despite such research, a comprehensive evaluation of the specific copy number thresholds associated with developmental and tissue-specific phenotypes, and the phenotypic consequences of high rDNA copy number have yet to be established in multicellular organisms.

### rDNA sequence variation

Intraindividual rDNA sequence polymorphism is commonly observed in eukaryotic organisms (Gribble and Anderson 2007; Simon and Weiß 2008; Bik *et al*. 2013; Wang *et al*. 2019; Stadler *et al*. 2020). The *D. pulex* rDNA in the current study contained 222 SNPs in the 10,363 nt included in the reference sequences. These occurred primarily in the rapidly evolving spacer regions, which is a consistent pattern among eukaryotic taxa (Ganley and Kobayashi 2007; Stage and Eickbush 2007; James *et al*. 2009). However, we also observed variation in the 28S gene. Most of the SNPs occurred in variable regions, but seven occurred in core regions. In their study of sequence variation in the rDNA of 12 individuals from different *Drosophila* species, Stage and Eickbush. (2007) also found that the expansion regions of the 18S and 28S genes exhibited more variation than the core regions. This was attributed to the A–T-rich sequences in the expansion regions, which can undergo rapid segmental changes (Hancock *et al*. 1988; Stage and Eickbush 2007).

The presence of SNPs in the expansion regions of rRNA genes suggests their tolerance for sequence variation, likely due to their lower significance for ribosome function compared to the core regions, which are highly conserved even among distantly related species (Stage and Eickbush 2007). Indeed, the mean population frequency of the most common variant at some of the thirty 28S SNPs we observed exceeded 20%, and these variants exceeded a frequency of 95% in some individuals, suggesting that they are neutral. Even if some of these variants are not completely neutral, natural selection only operates when deleterious variants reach a threshold frequency, below which they may have little or no impact on organism fitness (Stage and Eickbush 2007). We did not detect any variants in the 18S gene that met our criteria for retention. However, it is possible that some variants that did not meet these criteria were not PCR or sequencing artifacts, but were real variants present at very low frequency. This low-level genetic diversity is frequently observed in yeast strains from various geographic locations, with approximately 80% of variants occurring at a frequency of less than 10% within the rDNA (James *et al*. 2009).

The number of *D. pulex* SNPs and the frequency of variants at these sites varied substantially across individuals and populations. The fact that some variants, including some in the 28S gene, were found in eight or more populations suggests they have persisted for long periods of time despite concerted evolution. This is similar to the results of McTaggart and Crease (2005, 2009) who surveyed length variants in an expansion segment of the 18S gene in populations of *D. obtusa* from the Midwest United States and Crease (1995) who surveyed variation in the IGS subrepeat arrays in *D. pulex* individuals from BUS, KAP and PA. Variants found in only one population could have arisen in situ and have not yet spread via gene flow. On the other hand, some variants that were only present in two or three populations showed strong disequilibrium with each other and did not always occur in the populations that are closest to one another. For example, the variant alleles at 22 SNPs (5 in IGS1, 13 in IGS2, and 4 in the 28S gene) were only found in the same three populations (KAP, LPB, and PA) and were always found together in five of the 17 haplotypes in these populations (KAP3, KAP5, LPB4, LPB6, and PA5). The persistent strong linkage disequilibrium among variants across the entire rDNA repeat suggests that they did not arise independently in each population and recombination alone was insufficient to decrease this linkage disequilibrium. Moreover, it is noteworthy that the pattern of this robust disequilibrium facilitated the identification of a limited number of haplotypes in each population (see below).

Intraindividual variation in *Daphnia* rDNA was also observed by Ambrose and Crease (2011) who studied variation in the IGS sequences from 10 individuals representing four species in the *D. pulex* complex. They found that levels of sequence heterogeneity varied along the IGS with the lowest levels observed downstream of the 28S gene and the core promoter. They attributed differences within and between species to the interplay between recombination rates and selective constraints, resulting in unique evolutionary paths in different IGS regions.

### Haplotype variation

There was substantial haplotype diversity within populations with intraindividual variation exceeding variation among individuals in all populations except NFL and PA. This is likely due to the lower average heterozygosity in individuals from the latter two populations. Although average heterozygosity was lower in CHQ, individuals tended to have the same high-frequency haplotype, which decreased variation among individuals. Even so, except for CHQ, the same haplotype did not predominate in all individuals, leading to substantial differentiation among individuals within populations (*F*_SC_ values ranged from 0.305 to 0.638). These findings align with those of Crease and Lynch (1991) who analyzed restriction site variation in the rDNA of 90 *D. pulex* isolates from five populations in the Midwest United States, including three of the populations surveyed in this study (BUS, KAP, and PA). They identified 37 repeat types, four of which occurred in all populations, but also many others that occurred in only one population. Their estimates of *F*_SC_ ranged from 0.21 to 0.59, which is similar to our results. Although the frequency of the unique repeat types tended to be low, they were sometimes present in high frequency within an individual suggesting that repeat types spread more rapidly through rDNA arrays than the arrays carrying the variant spread through populations (Crease and Lynch 1991).

The correlation between H_e_ within individuals and the diploid number of 28S genes was significant and negative, suggesting that large rDNA arrays tend to have less variation than smaller ones. Indeed, one haplotype often predominated in individuals with the highest copy number in a population, while individuals with lower copy number had more haplotypes at intermediate frequencies. This suggests that variants tend to be clustered along rDNA arrays, which has been suggested previously (Crease 1995; McTaggart *et al*. 2007; Glass *et al*. 2008), and copy number increases often involve expansion of homogeneous stretches of repeats. This would also contribute to the variation in haplotype frequencies observed among individuals in a population. On the other hand, if individuals from BUS and PA were omitted from the analysis, there was no correlation, so overall, the level of haplotype variation within an rDNA array is not strongly influenced by its size. James *et al*. (2009) also found no correlation between the number of SNPs in individual *Saccharomyces cerevisiae* and rDNA copy number.

The pattern of high differentiation of haplotype frequencies among individuals is not consistent with the model of molecular drive proposed by Ohta and Dover (1984). They suggested that the frequency of a variant that is spreading throughout a sexual population will be similar among individuals because chromosomes carrying the variant will spread among individuals faster than the variant will spread within arrays via DNA turnover. This is clearly not the case in *D. pulex* rDNA, suggesting that there is ample opportunity for natural selection and genetic drift to act on this variation. Given that no one haplotype dominates within populations [except for CHQ, which has lower effective population size than the other populations according to Maruki *et al*. (2022)], it seems likely that haplotype frequencies in populations are most strongly affected by drift acting on the products of recombination within and between chromosomes.

In addition to substantial variation within populations, we also observed substantial haplotype differentiation among populations, with an *F*_CT_ value of 0.253. This value is lower than the overall value of *F*_SC_ (0.459), even though only one haplotype was shared among populations. However, haplotypes in different populations often have the same genotype at many SNPs that are shared among populations (e.g. KAP3, KAP5, LPB4, LPB6, PA5 discussed above), suggesting that there has been and continues to be some gene flow among populations.

Variation in copy number and the haplotype network, and analysis of complete IGS sequences (Ambrose and Crease 2011) make it clear that recombination creates variation in *Daphnia* rDNA, although it is not sufficient to eliminate linkage disequilibrium among SNPs. Changes in copy number generally involve unequal crossing over events that move blocks of rDNA repeats between sister chromatids and homologous chromosomes. However, such events only involve one or a few repeats in each partner, which may create new haplotypes without having any impact on the sequence of the other repeats in the rDNA array. In addition, recombination between haplotypes that currently do not occur in the same population, and new mutations continue to generate new haplotypes, which spread within populations faster than they spread among populations. Indeed, more than half of the SNPs we identified were unique to a single population and many of them accounted for differences between haplotypes both within and between populations.

Although the AMOVA identified significant differentiation among the 10 populations, the Mantel test suggested that there is no significant isolation by distance. This is consistent with the topology of the NJ tree generated from the pairwise estimates of *F*_CT_ in which populations that are geographically closest do not always cluster with one another. On the other hand, Maruki *et al*. (2022) estimated *F*_ST_ based on sequence variation in protein-coding genes and flanking regions in over 800 *D. pulex* individuals from 10 populations, (including the same genome sequences analyzed in the current study) and obtained a similar value of mean *F*_ST_ (∼0.27) after excluding sites with low-frequency alleles (the mean decreased to ∼0.13 when these sites were included). In contrast to our results, they did find that geographically close populations clustered together on a NJ tree based on pairwise *F*_ST_ estimates, and there was significant but weak isolation by distance. However, geographic distance only accounted for 20% of the variation in average pairwise *F*_ST_ values.

The lack of isolation by distance in rDNA is likely related to the different dynamics of allele frequency change in single-copy loci compared to a large multigene family. The strong pattern of linkage disequilibrium among rDNA SNPs that occurred in multiple populations suggests that this variation existed before the expansion of *D. pulex* northward following retreat of the last glaciers. This was also suggested for 18S length variants in *D. obtusa* from the Midwest United States (McTaggart and Crease 2009). Subsequent differentiation among populations occurred via genetic drift of ancestral haplotype frequencies both within chromosomes (rDNA copy number change) and among individuals. In addition, recombination and mutation subsequently created new haplotypes within populations as discussed above.

Other studies (Crease *et al*. 1990; Černý and Hebert 1993; Hebert and Finston 1996; Crease *et al*. 1997; Taylor *et al*. 1998; Lynch *et al*. 1999; Orsini *et al*. 2013; Fields *et al*. 2015) have found that gene flow among *Daphnia* populations is restricted on a local scale, but there is much less differentiation at the regional scale with little or no association between population differentiation and geographic distance. As much of North America and Europe were glaciated until about 20,000 years ago (Hewitt 2000), this pattern could be a consequence of persistent founder effects that occurred during postglacial colonization of new habitats by few propagules followed by rapid population expansion (Boileau *et al*. 1992). Boileau *et al*. (1992) argued that the substantial diversity among populations created by such founder effects could persist for thousands of generations if rates of gene flow are modest. Moreover, effective rates of gene flow could be lower than rates of dispersal because new colonists often fail to integrate into established populations due to priority effects and local adaptation (De Meester *et al*. 2002). In contrast, the lack of isolation by distance and low regional divergence has been attributed to long-distance dispersal of diapausing eggs by wind and waterfowl (Crease *et al*. 1997; Taylor *et al*. 1998; Weider *et al*. 1999).

## Conclusions

This is the first study of rDNA variation in natural populations of *Daphnia* where both sequence and copy number variation were examined in the same individuals. We found substantial copy number and sequence diversity among individuals both within and between populations. The presence of many sequence variants in multiple populations suggests they have persisted long-term despite concerted evolution. Moreover, strong linkage disequilibrium among variants allowed the identification of haplotypes in each population. Although there is evidence that recombination among haplotypes from different populations creates new haplotypes, it is not sufficient to eliminate linkage disequilibrium within populations. Estimating copy number and haplotype diversity in the same individuals showed that the level of intraindividual sequence variation is not strongly correlated with copy number, and supported the conclusion of previous studies that variants are clustered within rDNA arrays. Overall, the patterns of variation we observed suggest a complex evolutionary history of rDNA in *D. pulex*. Future research should explore the functional implications of rDNA copy number and sequence variation on organismal phenotypes.

## Supplementary Material

jkae105_Supplementary_Data

## Data Availability

Genome sequence data are available in the GenBank BioProject Database at https://www.ncbi.nlm.nih.gov/genbank/. The accession numbers are listed in Supplementary Table 1 in File S1. The scripts and codes used in our analyses are available on GitHub at https://github.com/elguweidi/Daphnia.pulex.natural.population.git Supplemental material available at G3 online.
