# Supplementary material for: Copy number and sequence variation in rDNA of Daphnia pulex from natural populations: insights from whole-genome sequencing
Source: G3 (Bethesda). 2024 May 21;14(7):jkae105. doi: 10.1093/g3journal/jkae105 (PMC11228840; doi:10.1093/g3journal/jkae105)
Supplement: jkae105_Supplementary_Data [file jkae105_supplementary_data.zip › File_S3_G3-2024-405073.pdf]

File S3. Analysis of the 16 exons chosen to represent single copy genes (SCG) in *Daphnia pulex* genomes

This document describes a comparison of mean read depth of the 160 exons originally identified in the *D. pulex* genome and the 16 exons ultimately chosen to represent Single Copy Genes.

As described in the Methods:

> *R was used to identify genes that occur in both the 12,295 Single Copy Genes (SCG) identified in Ensembl as well as the 716 single copy orthologs conserved across eukaryotic genomes as described in Colbourne et al. (2011). This comparison reduced our list to 256 SCG. A general transfer format (GTF) file containing all information needed to extract the exons from the SCG was downloaded from the Ensembl Metazoa database and linux was used to create a list of exons in the SCG. A Python code was created to identify exons from 300 to 1500 bp, which resulted in 228 exons. We retained the longest exon from each gene, which reduced the number to 167.*
>
> *To generate a set of exons that consistently gave similar read depths within samples, we chose the genomes of 20 individuals from natural populations of D. pulex (Table S1 in FileS1), and 8 samples from D. pulex mutation accumulation lines […]. The genome sequences of these 28 samples [one was ultimately dropped] were mapped to the 167 exon sequences as indicated above.* ***We measured the interquartile range (IQR) of mean read depth for the 167 exons in each genome and then counted the number of genomes in which an exon fell within the IQR. This identified a set of 16 exons that fell within the IQR in at least 20 of the 27 genomes (74%)****. The difference between the mean read depth of these 16 exons and the mean read depth of all exons within the IQR of a genome was less than 2 reads in all cases.*

After deciding to use this set of 16 exons, we wanted to know if they provided a reasonable representation of SCG read depth (**RD**) in a larger sample of the Daphnia genomes in our study. Thus, we chose 10 of the genomes from the original 20 from natural populations and then randomly chose an additional 40 genomes, spread across the 10 populations for a total of 50 genomes. We mapped all of them for the 167 exons identified previously. We found that 7 exons gave unusually high RD in the original analysis as well as the new samples, so we excluded them, leaving 160 exons.

To compare the distribution of RD of the 160 exons with those of the 16 chosen exons, we plotted a frequency histogram of RD for the former and overlaid the RD of each of the 16 exons on this plot (red dashed lines in plots at the end of this document). These plots are on the following pages of this document. We also added the mean RD of the 160 exons (mean160, green line) and the mean RD of the 16 exons (mean16, purple line) to the plots. In addition, we created a summary table of the results, which follows this text.

We also did a bootstrap analysis in which we randomly sampled 16 exons from the 160 with replacement to create 1000 bootstrap replicates for each genome. We calculated the mean16 of each replicate and plotted the results as a histogram. Not surprisingly, the mean of the 1000 mean16 replicates was identical to the original mean160 value.

In summary, the comparison of mean160 and mean16 for 50 genomes is as follows:

- The mean difference between mean160 and mean16 = 0.46 reads (max = 2.86, min = -1.06).
- The mean absolute difference between mean160 and mean16 = 0.84 reads (max = 2.86, min = 0.002).
- Mean160 > mean16 in 27 genomes (54%), mean160 < mean16 in 22 genomes (44%) suggesting there is no systematic bias across genomes. Moreover, the order of the 16 exons (red lines) on the plots differs between genomes (data not shown).
- The absolute value of 36 differences (72%) between mean160 and mean16 < 1.00 read.
- 13 of 14 differences > 1 read were positive (i.e. mean160 > mean16) suggesting that the unusually high outliers in the 160 were primarily responsible for the largest differences between the 2 sets of exons.
- The absolute value of 26 differences (52%) < 0.50 read.
- The absolute value of 9 differences (18%) >1.50 reads. All but 1 of these was an individual in BUS or PA (we discuss the unusually high rDNA copy number in these populations in response to another comment).
- The distribution of RD values for the 16 exons (red dashed lines) within genomes do not occur in the extreme tails of the distribution in any of the genomes we analyzed.

We concede that it is not possible to find a set of SCG benchmark exons whose mean RD is within a very limited range in every genome. However, it is not feasible to choose a unique set of exons for every genome, and we suggest that the 16 exons we have chosen provide a reasonable benchmark of RD of SCGs in the *D. pulex* genome. Moreover, they provide rDNA copy numbers that are very similar (except for PA) to those estimated using qPCR (eg. Eagle and Crease 2012, 2016; Harvey et al. 2020).

Table S1. Summary of results comparing read depth of 160 SC exons and and a subset of 16 exons in genomes from natural populations of *Daphnia pulex*. The results presented below are based on the results of mapping all 160 exons in the same mapping analysis. The estimates of haploid 18S copy number are based on the 16 chosen exons.

genome	individual	mean of 160 exons	mean of 16 exons	mean160 - mean16	|mean160 - mean16|	haploid 18S copy number
SRR7589089	BUS02	18.55	17.24	1.31	1.31	797
SRR7589090	BUS03	13.02	11.04	1.98	1.98	1548
SRR7589138	BUS11	10.72	8.46	2.26	2.26	512
SRR7589142	BUS12	10.23	11.20	-0.97	0.97	402
SRR7589143	BUS13	9.38	9.90	-0.52	0.52	398
SRR7589150	BUS14	15.21	14.68	0.53	0.53	355
SRR7589151	BUS15	5.17	6.04	-0.87	0.87	460
SRR7589155	BUS16	10.86	9.76	1.10	1.10	575
SRR7589175	BUS17	16.46	16.46	0.00	0.00	282
SRR7592625	CHQ01	23.40	23.67	-0.27	0.27	394
SRR7592631	CHQ05	21.15	19.89	1.26	1.26	475
SRR7592632	CHQ06	20.27	19.83	0.44	0.44	800
SRR7592648	CHQ13	19.69	19.25	0.43	0.43	473
SRR7592689	CHQ15	24.03	23.30	0.73	0.73	387
SRR7592693	CHQ16	22.05	23.03	-0.98	0.98	359
SRR7592705	CHQ17	19.21	19.69	-0.48	0.48	370
SRR7591941	EB02	11.53	11.67	-0.14	0.14	377
SRR7591975	EB14	22.12	22.35	-0.23	0.23	263
SRR8401654	KAP01	24.17	24.53	-0.36	0.36	323
SRR8401684	KAP02	18.92	19.73	-0.81	0.81	179
SRR8401685	KAP03	23.08	22.60	0.48	0.48	336
SRR8401686	KAP04	20.28	19.65	0.63	0.63	211
SRR8401687	KAP05	22.02	22.30	-0.28	0.28	217
SRR8401688	KAP06	21.37	20.94	0.43	0.43	249
SRR8401689	KAP07	11.49	11.91	-0.42	0.42	388
SRR7592102	LPA09	15.88	15.85	0.03	0.03	328

SRR7592105	LPA11	17.72	15.92	1.80	1.80	349
SRR7592394	LPB02	36.41	35.95	0.46	0.46	503
SRR7592395	LPB03	26.13	26.58	-0.45	0.45	315
SRR7592738	NFL03	28.71	28.39	0.31	0.31	422
SRR8401691	NFL17	17.04	17.05	-0.01	0.01	265
SRR8401692	NFL18	19.04	19.12	-0.08	0.08	348
SRR8401693	NFL19	26.00	26.31	-0.31	0.31	356
SRR8401694	NFL20	8.25	8.31	-0.06	0.06	272
SRR8401695	NFL21	7.96	7.65	0.31	0.31	367
SRR8401696	NFL22	24.14	25.20	-1.06	1.06	288
SRR8401697	NFL23	16.62	16.44	0.18	0.18	321
SRR7594196	PA05	8.65	6.02	2.63	2.63	2180
SRR7594197	PA06	17.66	15.33	2.33	2.33	1264
SRR7594210	PA08	6.73	5.48	1.25	1.25	1341
SRR7594215	PA09	11.44	8.58	2.86	2.86	1136
SRR7594219	PA10	10.86	8.42	2.44	2.44	1057
SRR7594235	PA13	10.32	7.80	2.52	2.52	866
SRR7594253	PA14	11.56	8.99	2.57	2.57	825
SRR12689207	POV01	21.20	20.45	0.75	0.75	231
SRR12689208	POV02	20.04	20.13	-0.09	0.09	278
SRR7593845	TEX04	10.85	10.98	-0.13	0.13	312
SRR7593846	TEX05	20.01	20.35	-0.34	0.34	501
SRR7593906	TEX17	18.26	18.78	-0.52	0.52	670
SRR7593907	TEX18	10.10	9.74	0.36	0.36	426
**mean**		**17.12**	**16.66**	**0.46**	**0.84**	
**max**		**36.41**	**35.95**	**2.86**	**2.86**	
**min**		**5.17**	**5.48**	**-1.06**	**0.00**	

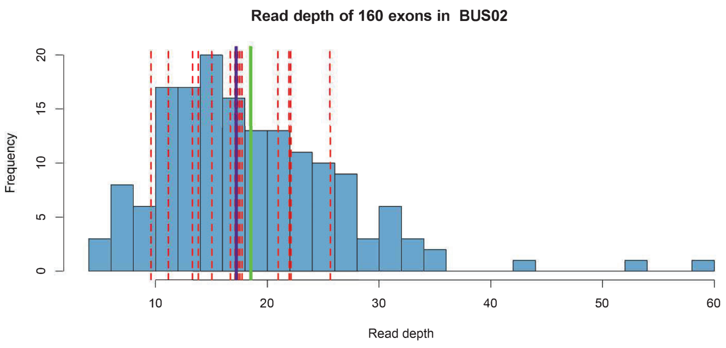
Read depth of 160 exons in BUS02
Frequency
Read depth

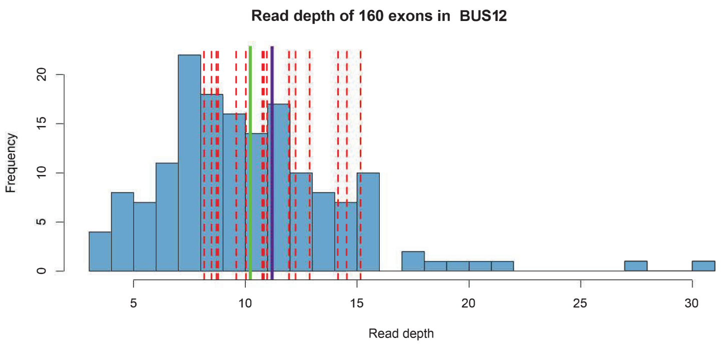
Read depth of 160 exons in BUS12
Frequency
Read depth

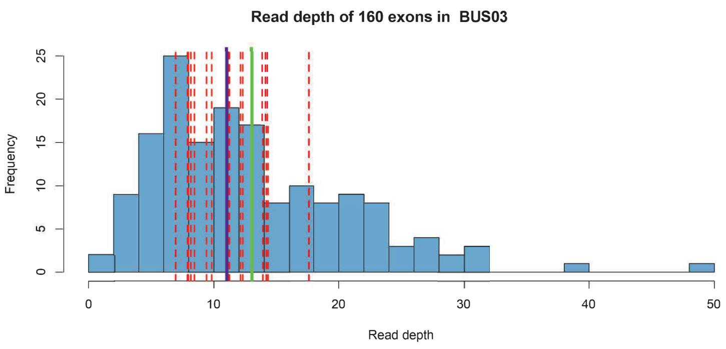
Read depth of 160 exons in BUS03
Frequency
Read depth

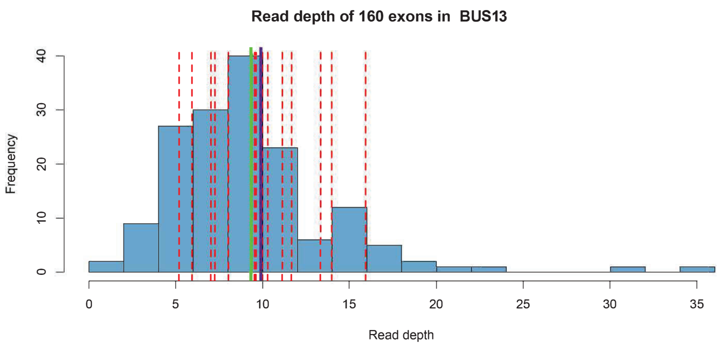
Read depth of 160 exons in BUS13
Frequency
Read depth

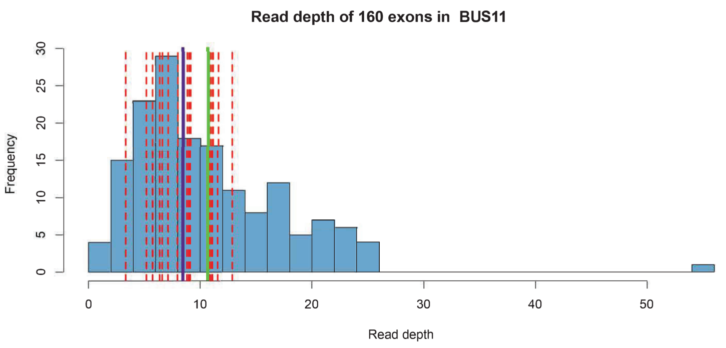
Read depth of 160 exons in BUS11
Frequency
Read depth

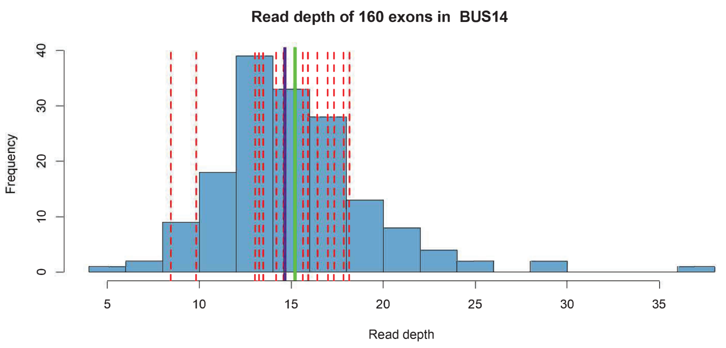
Read depth of 160 exons in BUS14
Frequency
Read depth

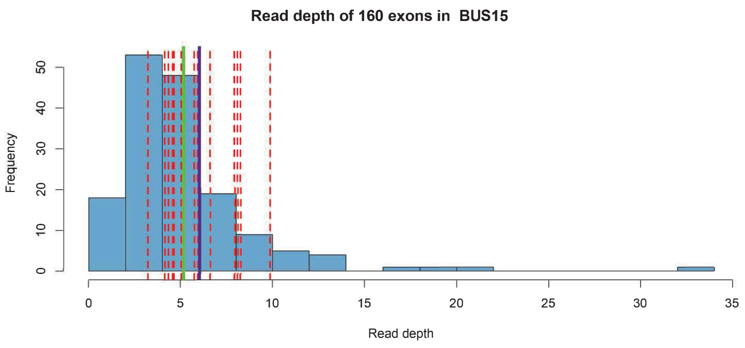
Read depth of 160 exons in BUS15
Frequency
Read depth
0 5 10 15 20 25 30 35
0 10 20 30 40 50

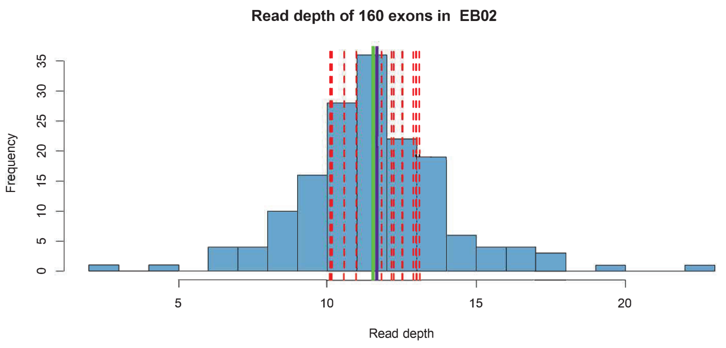
Read depth of 160 exons in EB02
Frequency
Read depth
5 10 15 20
0 5 10 15 20 25 30 35

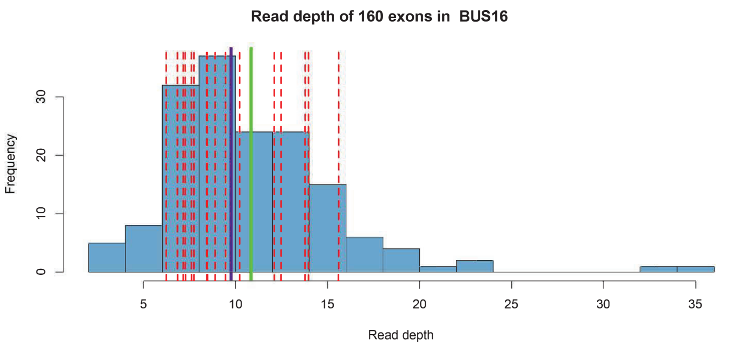
Read depth of 160 exons in BUS16
Frequency
Read depth
5 10 15 20 25 30 35
0 10 20 30

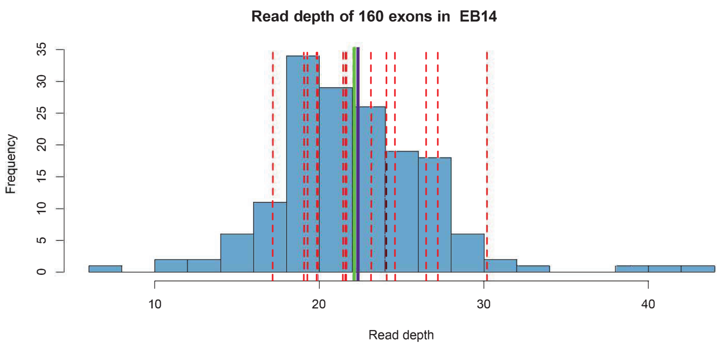
Read depth of 160 exons in EB14
Frequency
Read depth
10 20 30 40
0 5 10 15 20 25 30 35

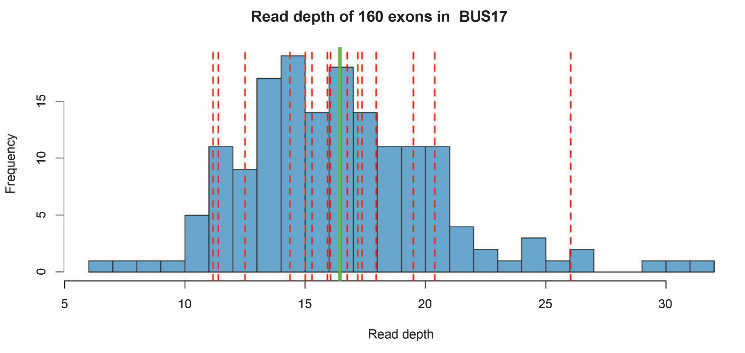
Read depth of 160 exons in BUS17
Frequency
Read depth
5 10 15 20 25 30
0 5 10 15

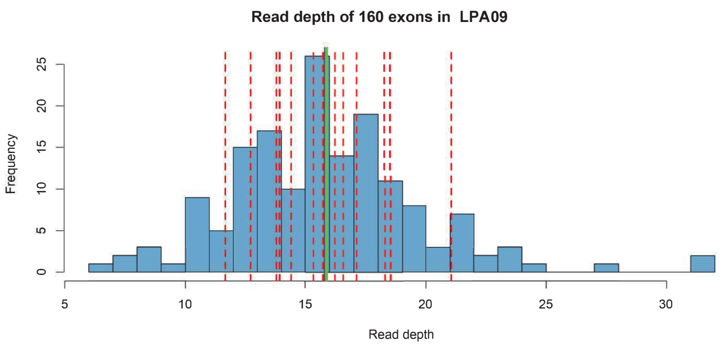
Read depth of 160 exons in LPA09
Frequency
Read depth
5 10 15 20 25 30
0 5 10 15 20 25

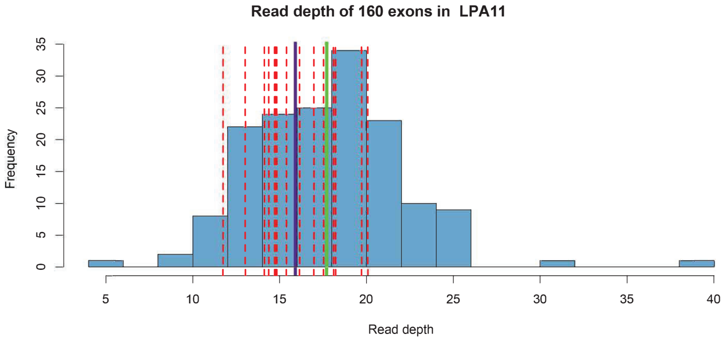
Read depth of 160 exons in LPA11
Frequency
Read depth

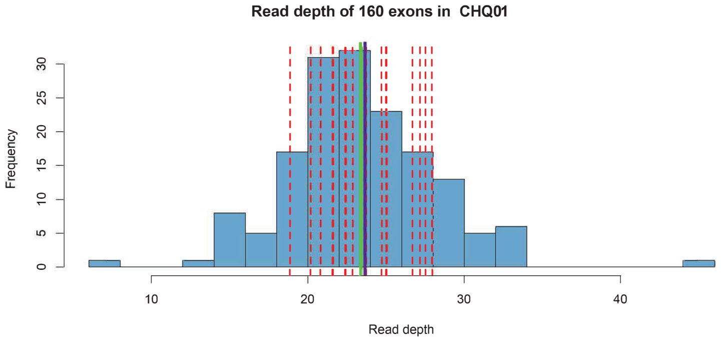
Read depth of 160 exons in CHQ01
Frequency
Read depth

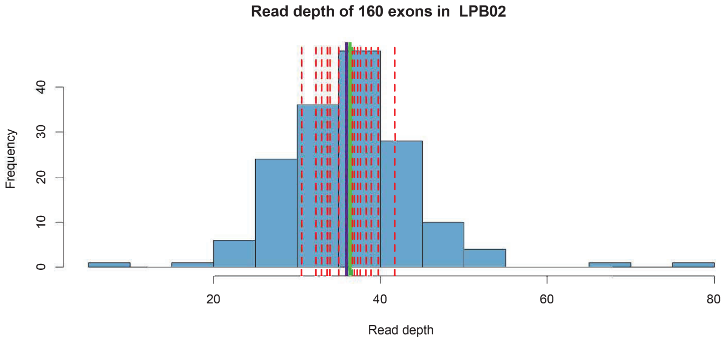
Read depth of 160 exons in LPB02
Frequency
Read depth

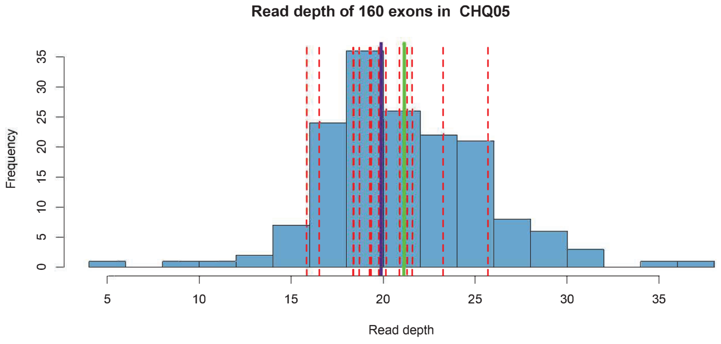
Read depth of 160 exons in CHQ05
Frequency
Read depth

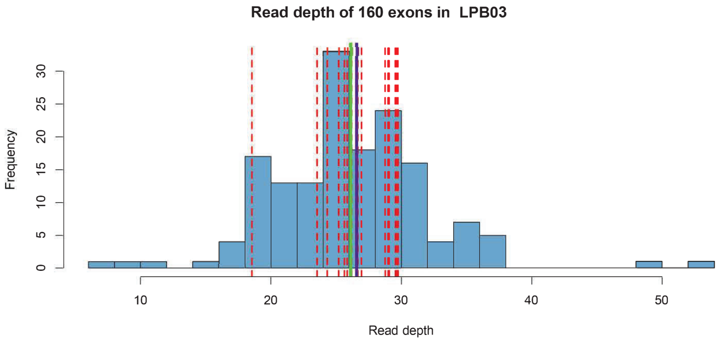
Read depth of 160 exons in LPB03
Frequency
Read depth

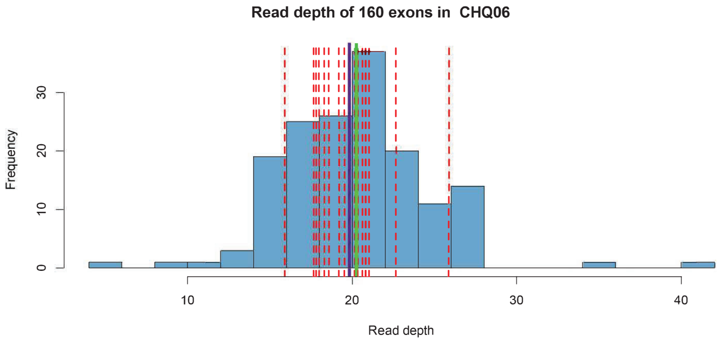
Read depth of 160 exons in CHQ06
Frequency
Read depth

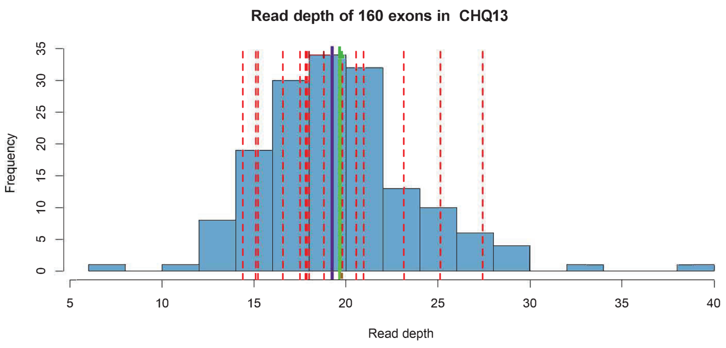
Read depth of 160 exons in CHQ13
Frequency
Read depth

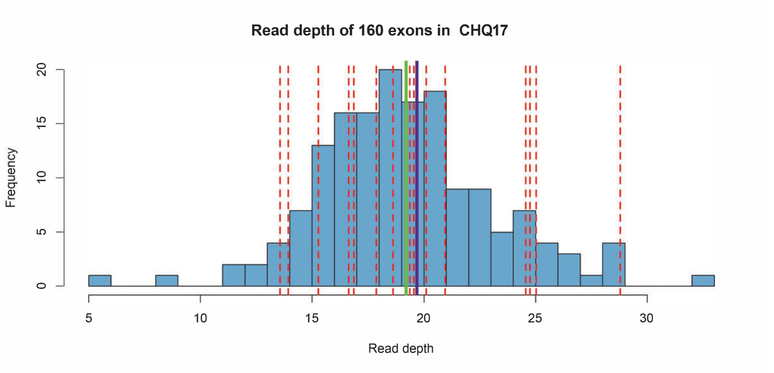
Read depth of 160 exons in CHQ17
Frequency
Read depth

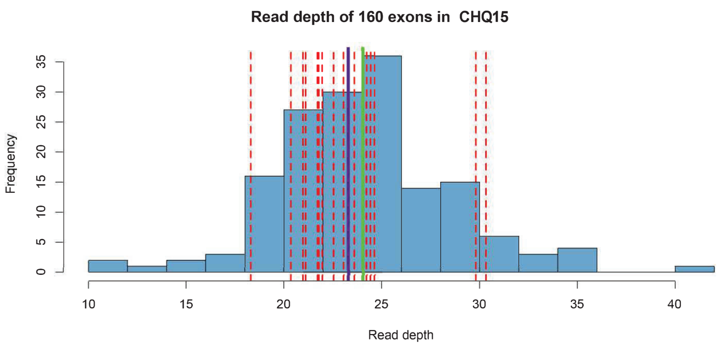
Read depth of 160 exons in CHQ15
Frequency
Read depth

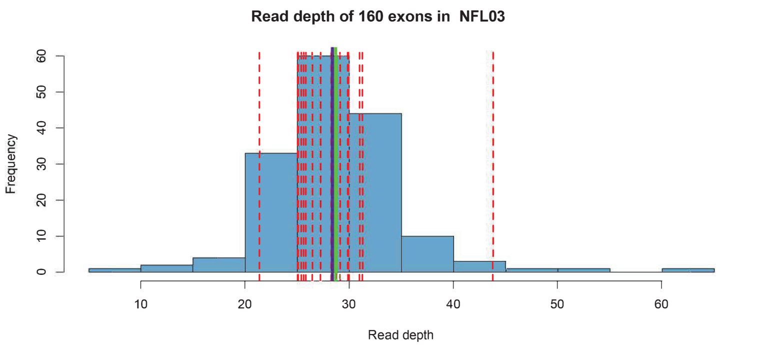
Read depth of 160 exons in NFL03
Frequency
Read depth

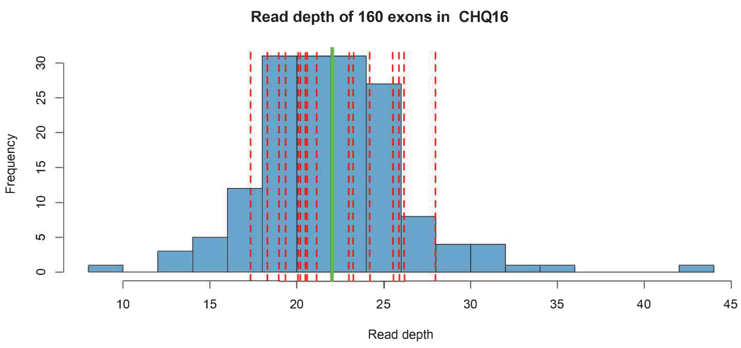
Read depth of 160 exons in CHQ16
Frequency
Read depth

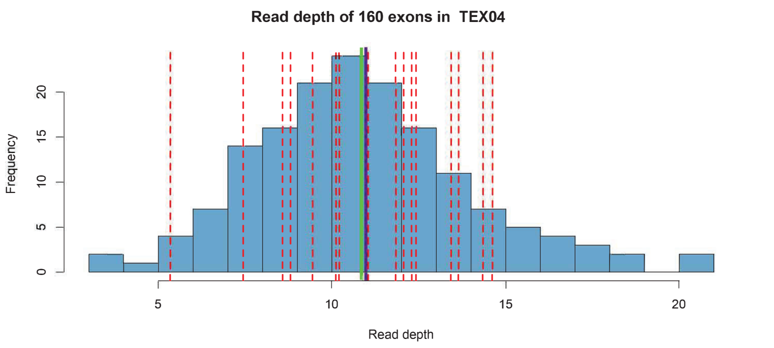
Read depth of 160 exons in TEX04
Frequency
Read depth

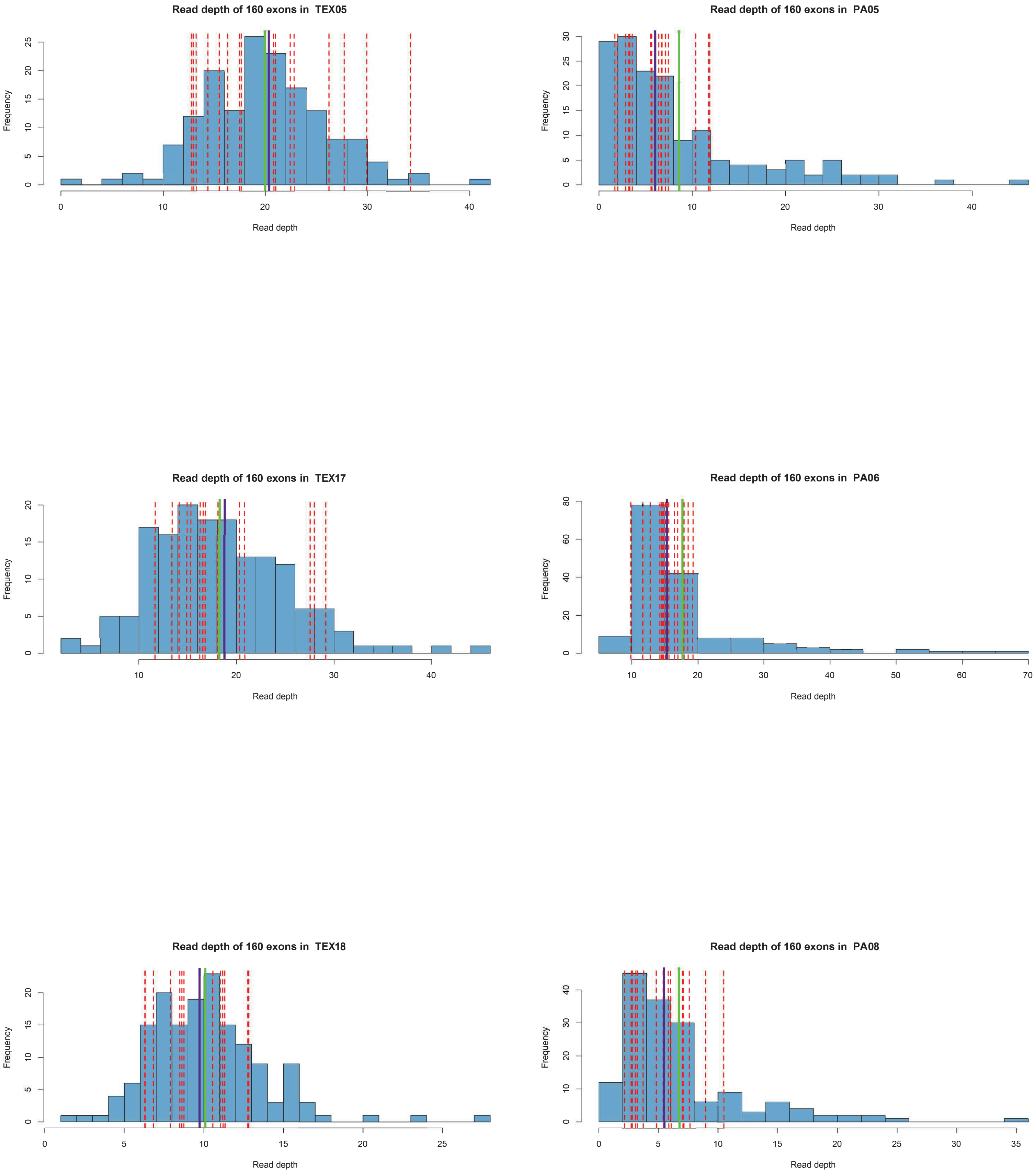
Read depth of 160 exons in TEX05
Read depth of 160 exons in PA05
Read depth of 160 exons in TEX17
Read depth of 160 exons in PA06
Read depth of 160 exons in TEX18
Read depth of 160 exons in PA08
Frequency
Read depth

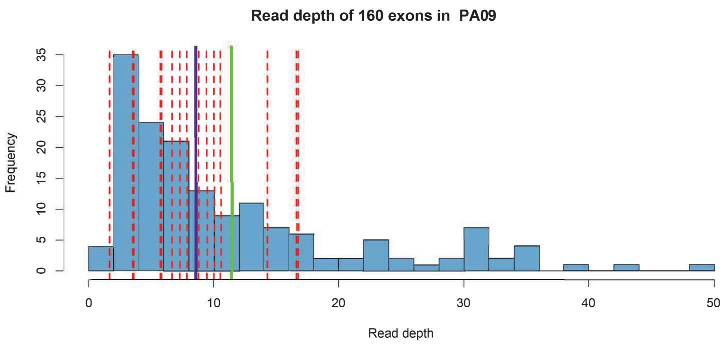
Read depth of 160 exons in PA09
Frequency
0 5 10 15 20 25 30 35
0 10 20 30 40 50
Read depth

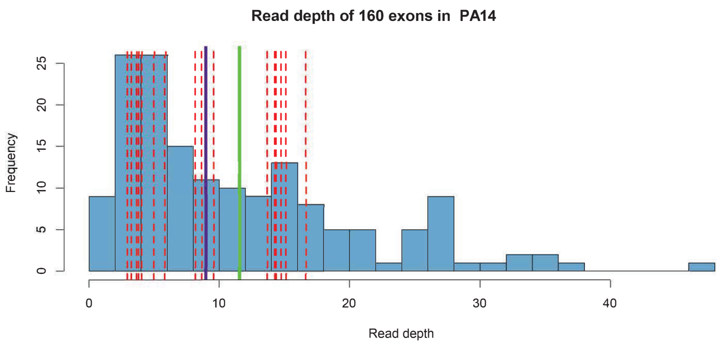
Read depth of 160 exons in PA14
Frequency
0 5 10 15 20 25
0 10 20 30 40
Read depth

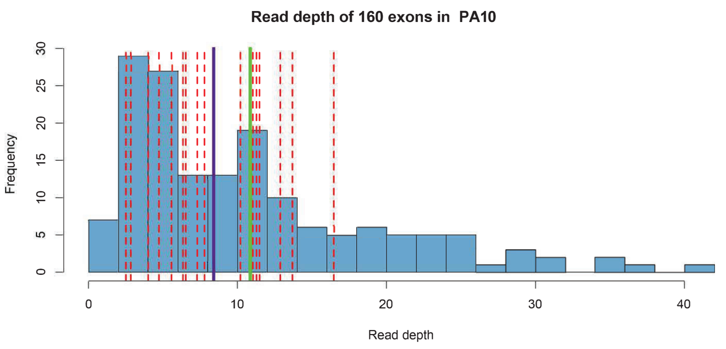
Read depth of 160 exons in PA10
Frequency
0 5 10 15 20 25 30
0 10 20 30 40
Read depth

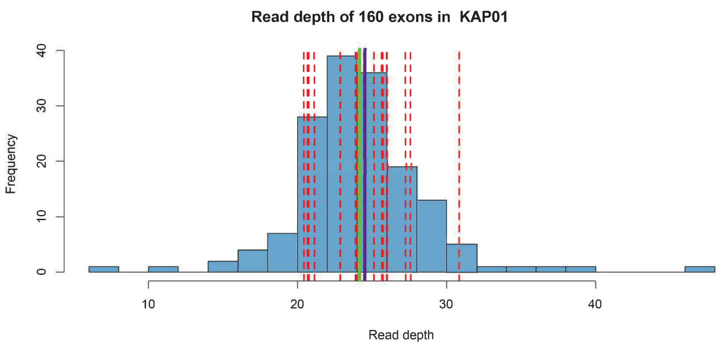
Read depth of 160 exons in KAP01
Frequency
0 10 20 30 40
10 20 30 40
Read depth

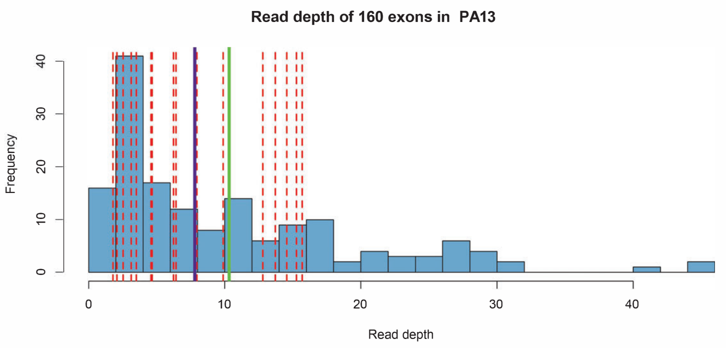
Read depth of 160 exons in PA13
Frequency
0 10 20 30 40
0 10 20 30 40
Read depth

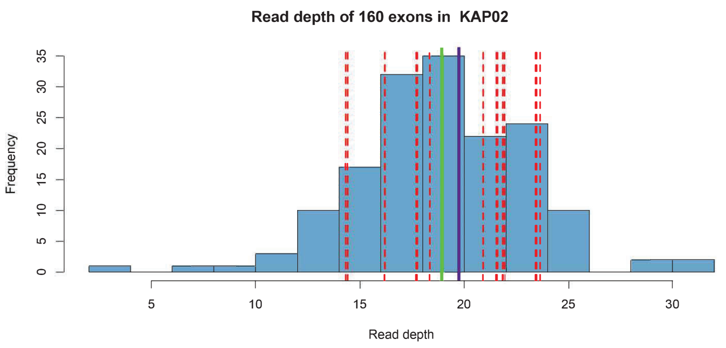
Read depth of 160 exons in KAP02
Frequency
0 5 10 15 20 25 30 35
5 10 15 20 25 30
Read depth

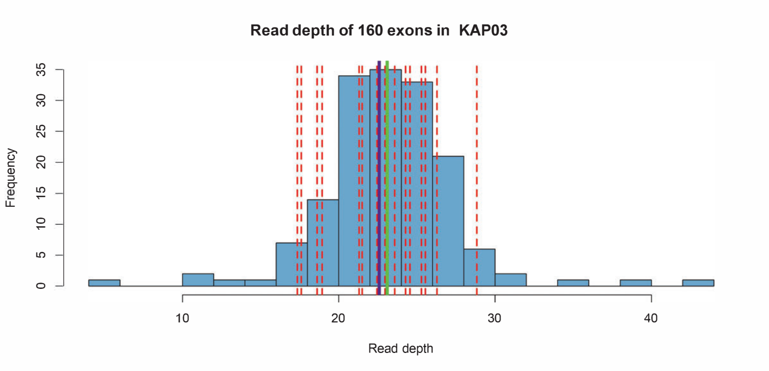
Read depth of 160 exons in KAP03
Frequency
0 5 10 15 20 25 30 35
10 20 30 40
Read depth

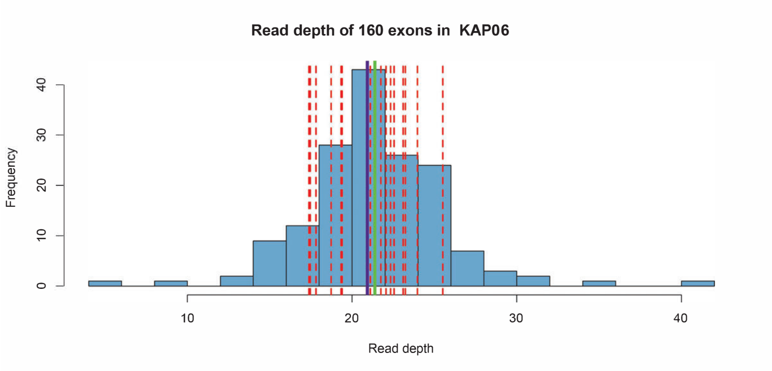
Read depth of 160 exons in KAP06
Frequency
0 10 20 30 40
10 20 30 40
Read depth

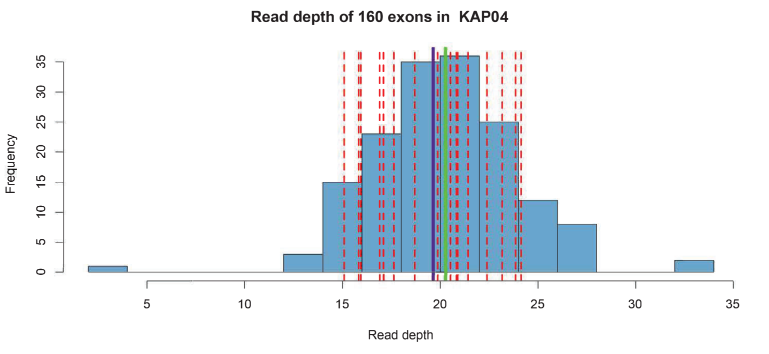
Read depth of 160 exons in KAP04
Frequency
0 5 10 15 20 25 30 35
5 10 15 20 25 30 35
Read depth

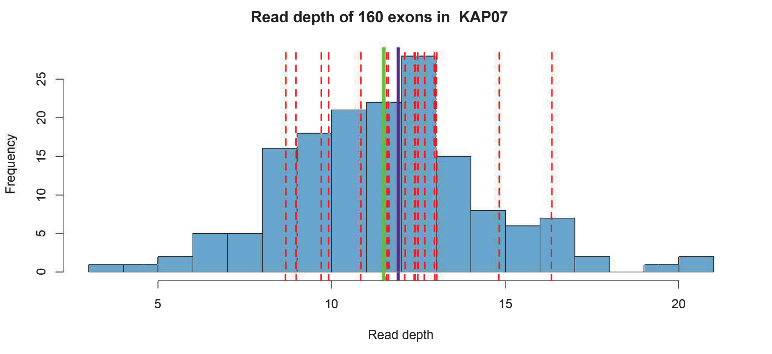
Read depth of 160 exons in KAP07
Frequency
0 5 10 15 20 25
5 10 15 20
Read depth

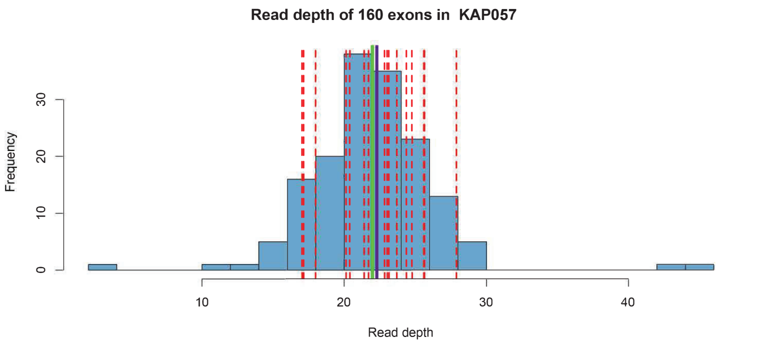
Read depth of 160 exons in KAP057
Frequency
0 10 20 30
10 20 30 40
Read depth

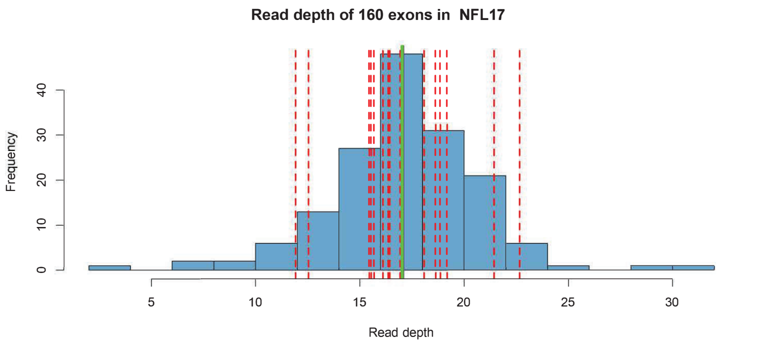
Read depth of 160 exons in NFL17
Frequency
0 10 20 30 40
5 10 15 20 25 30
Read depth

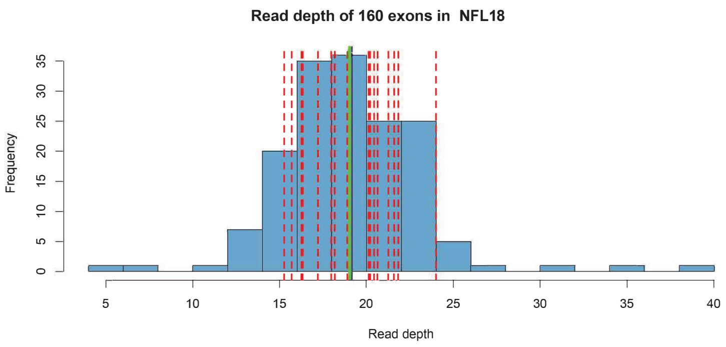
Read depth of 160 exons in NFL18
Frequency
Read depth
0 5 10 15 20 25 30 35
5 10 15 20 25 30 35 40

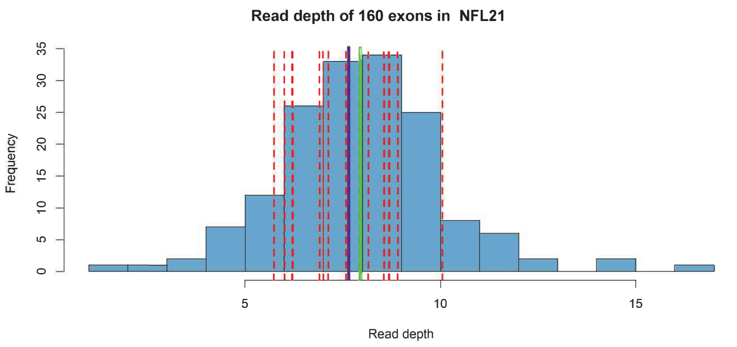
Read depth of 160 exons in NFL21
Frequency
Read depth
0 5 10 15 20 25 30 35
5 10 15

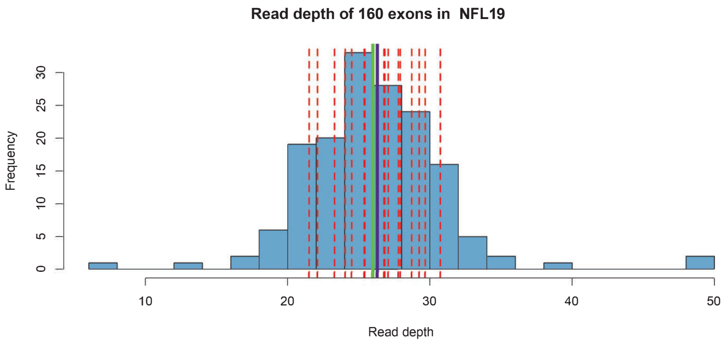
Read depth of 160 exons in NFL19
Frequency
Read depth
0 5 10 15 20 25 30
10 20 30 40 50

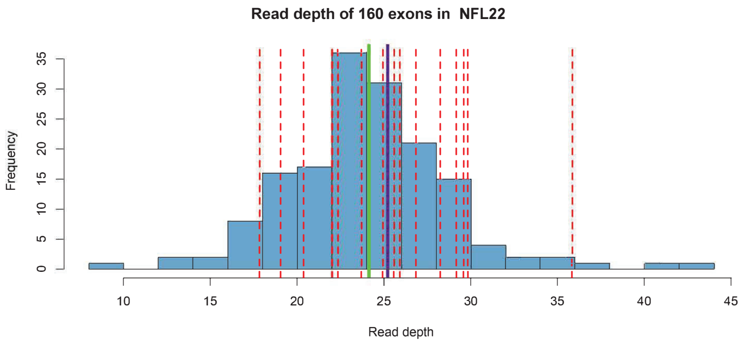
Read depth of 160 exons in NFL22
Frequency
Read depth
0 5 10 15 20 25 30 35
10 15 20 25 30 35 40 45

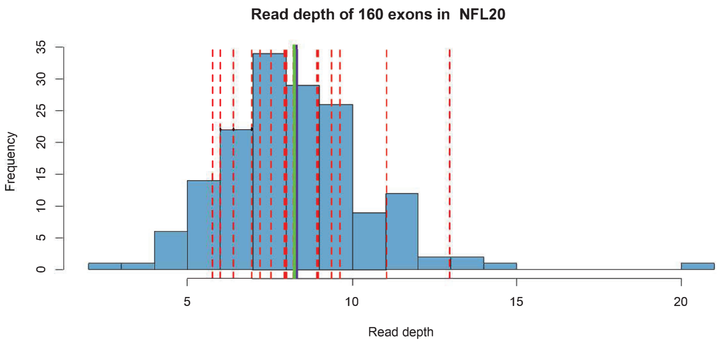
Read depth of 160 exons in NFL20
Frequency
Read depth
0 5 10 15 20 25 30 35
5 10 15 20

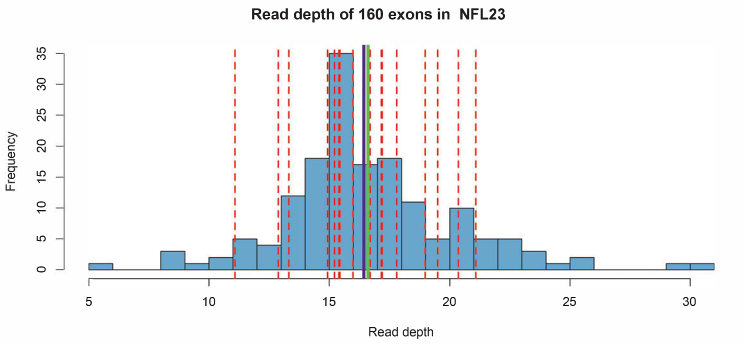
Read depth of 160 exons in NFL23
Frequency
Read depth
0 5 10 15 20 25 30 35
5 10 15 20 25 30

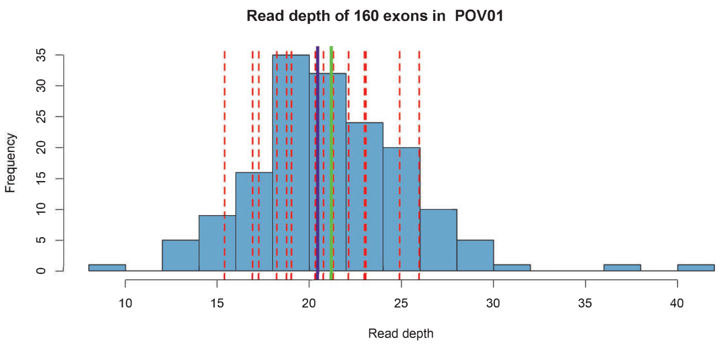
Read depth of 160 exons in POV01
Frequency
Read depth
0
5
10
15
20
25
30
35
10
15
20
25
30
35
40

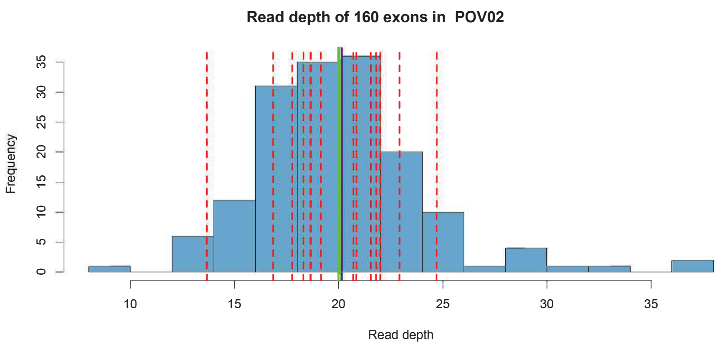
Read depth of 160 exons in POV02
Frequency
Read depth
0
5
10
15
20
25
30
35
10
15
20
25
30
35
