# Supplementary material for: Copy number and sequence variation in rDNA of Daphnia pulex from natural populations: insights from whole-genome sequencing
Source: G3 (Bethesda). 2024 May 21;14(7):jkae105. doi: 10.1093/g3journal/jkae105 (PMC11228840; doi:10.1093/g3journal/jkae105)
Supplement: jkae105_Supplementary_Data [file jkae105_supplementary_data.zip › File_S4_G3-2024-405073.pdf]

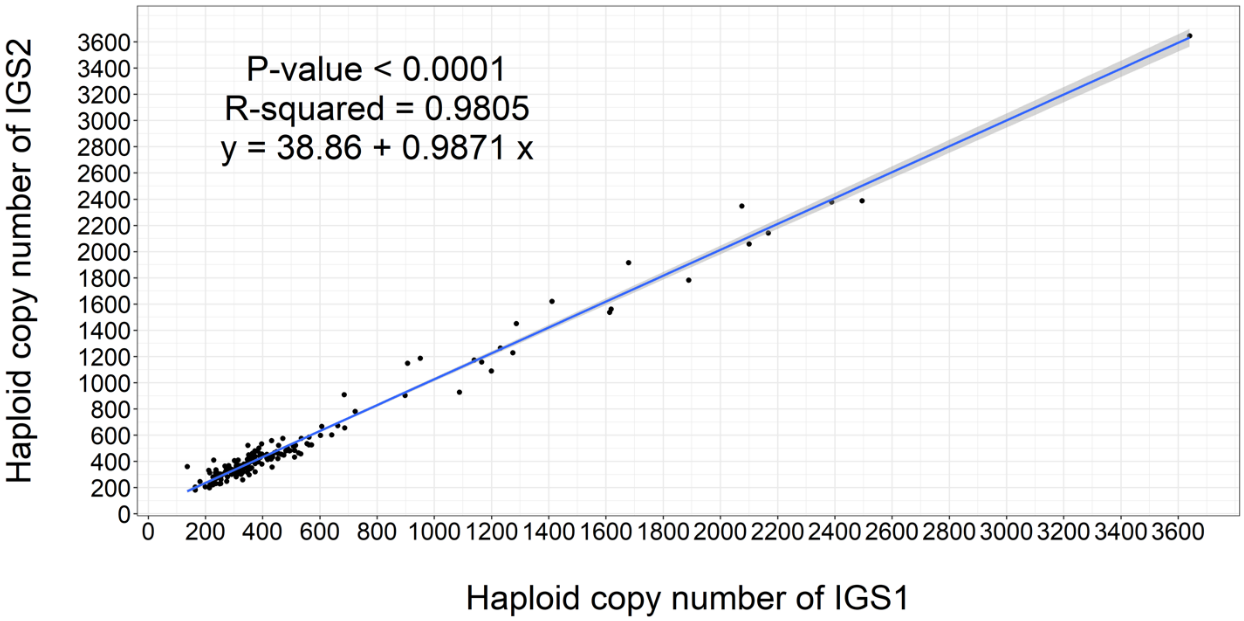

Figure S1. Linear regression of haploid copy number of IGS1 and IGS2. The gray shading indicates the 95% confidence interval.

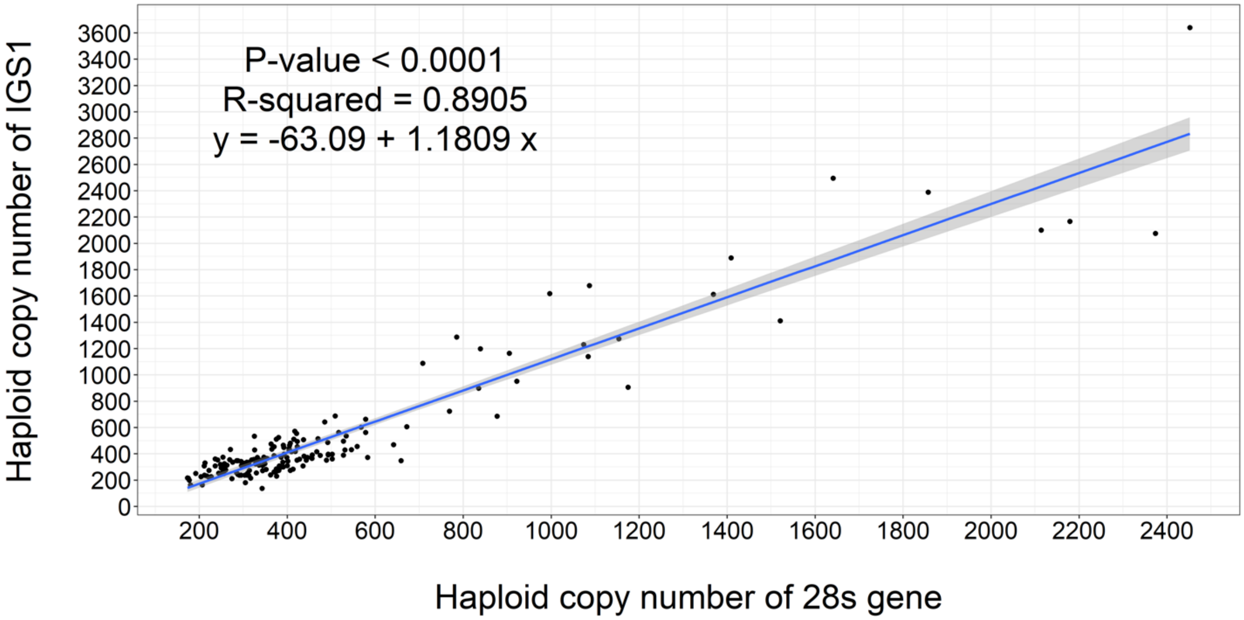

Figure S2. Linear regression of haploid copy number of the 28S gene and IGS1. The gray shading indicates the 95% confidence interval.

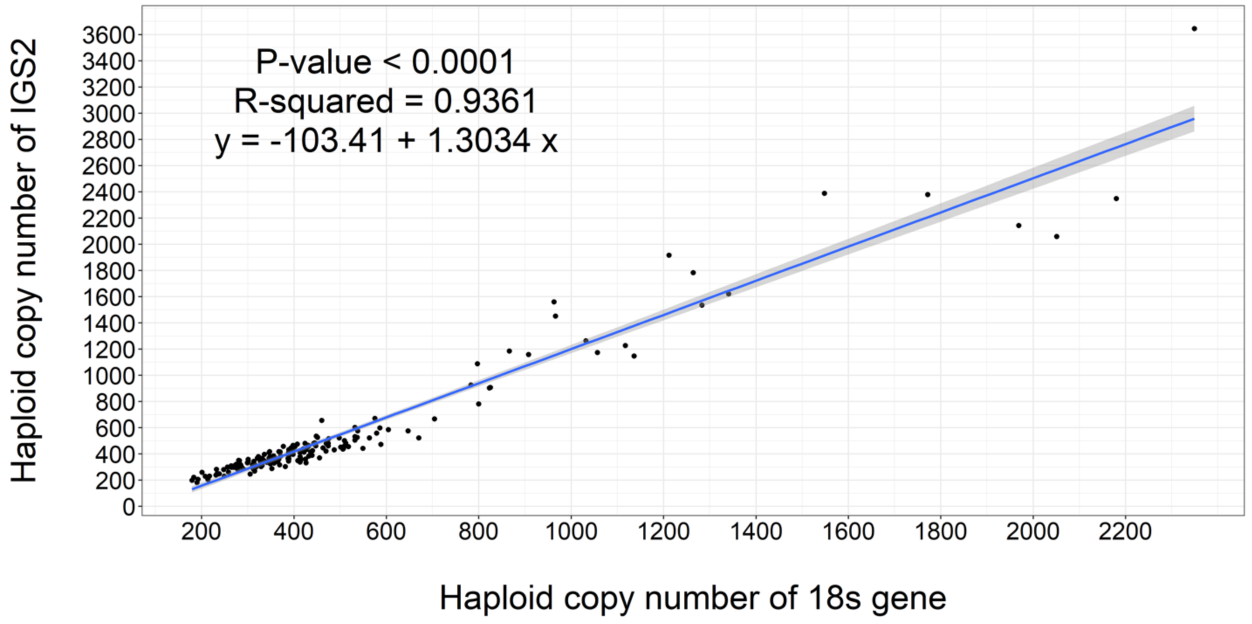

Figure S3. Linear regression of haploid copy number of the 18S gene and IGS2. The gray shading indicates the 95% confidence interval.

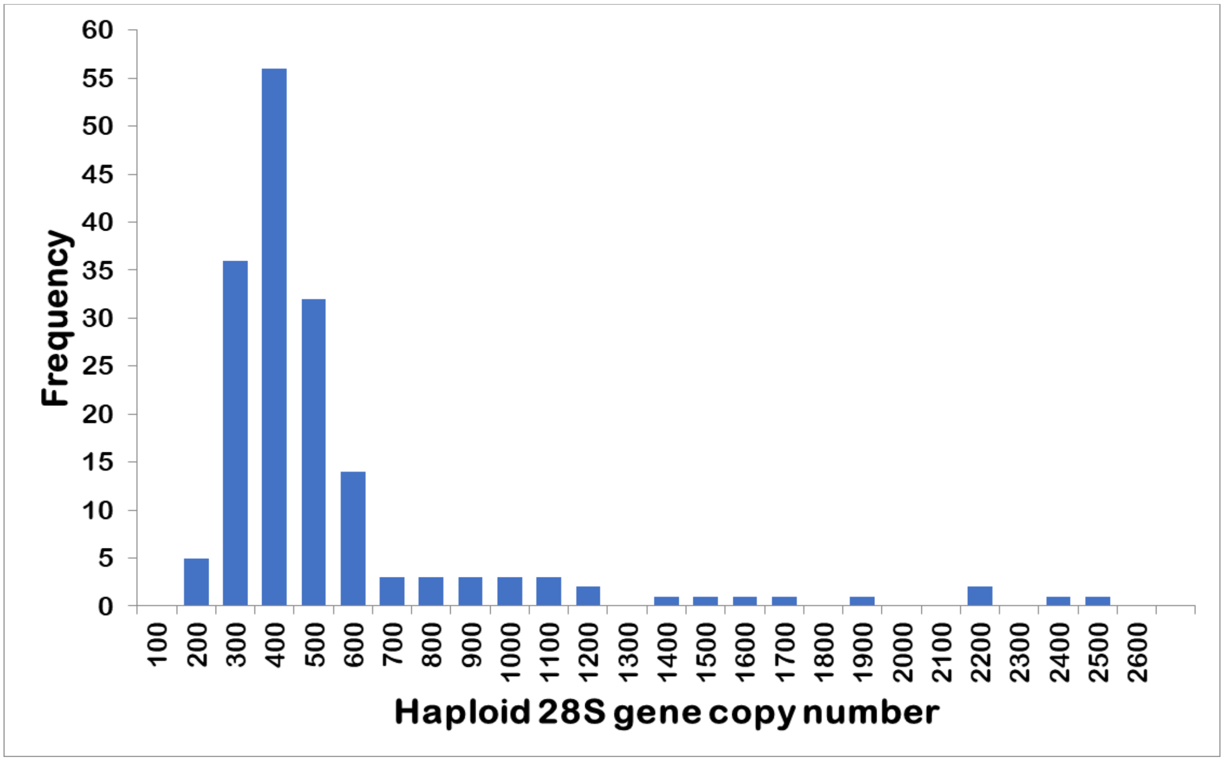

Figure S4. Frequency distribution of haploid 28S copy number in 169 individuals of *Daphnia pulex* from 10 natural populations. All values above 1000 occurred in individuals from population BUS or PA.
